# Multi-Model Approaches Reveal Cascading Regulation and Ecological Responses of Zooplankton to Seasonal and Water Quality Variations in Urbanized Rivers

**DOI:** 10.3390/biology15141211

**Published:** 2026-07-22

**Authors:** Jiawei Wu, Maojin Huang, Xue Yang, Baoshan Zhang, Siyao Liu, Lei Jian, Fei Xu

**Affiliations:** 1 College of Environmental Science and Engineering, China West Normal University, Nanchong 637009, China; 2 Sichuan Academy of Eco-Environmental Sciences, Chengdu 610046, China

**Keywords:** Jialing River Basin, zooplankton, aquatic ecological assessment, PLS-PM, community assembly mechanisms

## Abstract

Urbanization imposes multiple environmental stressors on urban rivers. Zooplankton are sensitive to environmental disturbances, making them useful tools for assessing ecological health and revealing river degradation processes. We surveyed the urban reaches of the middle and lower Jialing River and its tributary (Xichong River) during autumn 2022 and spring 2023. Water ecological status was assessed using the water quality index and zooplankton-based Index of Biotic Integrity. Community assembly mechanisms and their driving pathways were analyzed by combining the neutral community model (NCM, MST), random forest (RF), and partial least squares path modeling (PLS-PM). Key results: (1) The zooplankton community in the Jialing River Basin exhibited significant spatiotemporal heterogeneity. (2) The overall water ecological status was better in the mainstream than in the tributary, and better in autumn than in spring. (3) Stochastic processes dominated the Jialing River mainstream in autumn (MST = 0.5388), whereas deterministic processes dominated in all other habitats. (4) Seasonal variation was the key driver of community assembly (*R*^2^ = 0.253, *p* < 0.001), and COD_Mn_ was the primary water quality factor (IncMSE = 25.48%). In summary, multiple models combined quantified the dominant pathways of ecological response, providing theoretical support for the health management of urbanized rivers.

## Simple Summary

Rapid urbanization is threatening the health of urban river ecosystems. To address this challenge, managers first need accurate tools to assess ecological conditions. Zooplankton are tiny aquatic animals highly sensitive to environmental changes, and their community characteristics can effectively indicate the true ecological state of rivers. This study surveyed the mainstream of the Jialing River and its tributary, the Xichong River, during autumn 2022 and spring 2023. We analyzed zooplankton community composition and structure, combined with water quality measurements, to evaluate river health comprehensively, and quantified the influence of environmental factors on community changes. The ecological condition of the basin ranked as follows: autumn mainstream > spring mainstream > autumn tributary > spring tributary. Seasonal change was the most important factor affecting zooplankton communities. The mainstream in autumn was dominated by random factors, while environmental factors played a dominant role in the other three habitats. This study provides a scientific method for accurately assessing ecological conditions and identifying key influencing factors, offering theoretical support for ecological restoration and refined management of urban rivers in the upper Yangtze River Basin.

## 1. Introduction

Zooplankton serve as key bioindicators in aquatic ecosystems [1]. Compared to bioassessment frameworks centered on fish or benthic macroinvertebrates, zooplankton respond more rapidly to water environmental disturbances, thereby avoiding a lag in assessment status relative to actual environmental perturbations [2]. To date, zooplankton-based assessment methods have been applied in ecological monitoring [3]. Numerous studies have employed traditional multivariate ordination models such as redundancy analysis (RDA) and canonical correspondence analysis (CCA) to perform simple correlative fitting between the overall structure of zooplankton communities and environmental gradients [4]. Although such single-model analyses can preliminarily reflect the response of biological communities to environmental factors, they struggle to finely resolve the multi-layered regulatory relationships between community structure and complex composite environmental drivers, nor can they readily achieve refined and quantitative diagnosis of water environmental status [5,6]. This limitation hinders the in-depth dissection of the driving mechanisms underlying zooplankton communities and impedes the future development of more accurate predictive assessment tools for water ecological evaluation [7].

River ecosystems worldwide are facing habitat degradation and loss of aquatic ecological functions driven by human activities [2,8]. As the largest river basin in China, the Yangtze River Basin has experienced substantial deterioration of its aquatic ecosystems [9]. However, studies on the ecosystem health of this basin have mostly focused on the whole basin scale or on the middle and lower reaches [10], leaving relatively insufficient attention to key tributaries in the upper reaches. The Jialing River, the largest first-order tributary of the upper Yangtze River and flowing through the important urban agglomerations of Sichuan and Chongqing, holds an irreplaceable ecological strategic position [11]. In recent years, although water quality in the mainstream of the Jialing River has shown an improving trend, with our field measurements indicating long-term stable Class II water quality in the study area, ecological health problems in some of its typical urbanized tributaries still urgently require improvement. The Xichong River, a first-order tributary of the middle Jialing River, suffers from water resource scarcity and continuous inputs of pollution from both adjacent urban areas and non-point sources. Our water quality data show that most sampling sites fell into Class III–IV during both spring and autumn, markedly inferior to the mainstream, indicating that the aquatic ecological health status remains critical [12].

Scientifically robust water ecological assessment is a core tool for identifying the health status of aquatic ecosystems. Currently, water ecological assessment has shifted from an early focus on physicochemical parameters of water quality (e.g., DO, COD, pH, TP, TN) towards integrated evaluation systems incorporating measurable indices such as WQI and IBI [13]. In this transition, biological monitoring has gradually developed into a key approach comparable to physicochemical water quality assessment, owing to its unique advantage in capturing the cumulative historical effects of communities [14]. Concurrently, advances in mathematical modeling have provided effective tools for disentangling complex ecological relationships [15]. The neutral community model (NCM), based on the assumption of ecological equivalence among individuals of different species, allows for a qualitative assessment of whether community assembly deviates significantly from neutral expectations [16]. To overcome the limitations of qualitative judgments, Ning et al. (2019) [17] proposed the normalized stochasticity ratio (NST) and its variant, the modified stochasticity ratio (MST). This index quantifies the relative importance of stochastic versus deterministic processes using a threshold of 50%—a value derived from the null expectation of the neutral model, which assumes equal contributions of stochastic and deterministic processes under purely neutral dynamics. Random forest (RF), an ensemble learning algorithm, can evaluate variable importance to quantify the relative explanatory contribution of each environmental factor to the response variable [18]. Building on this, partial least squares path modeling (PLS-PM) integrates principal component analysis and multiple linear regression, enabling causal inference and path analysis under conditions of non-normally distributed data, a large number of indicators, and limited sample sizes [19]. These methods have been applied and validated in studies on plankton community assembly [20,21,22]. However, studies that integrate these approaches for water ecological assessment in the Jialing River Basin remain scarce.

Accordingly, this study took the Nanchong section of the Jialing River Basin as the study area. The sampling sites were classified into four groups according to season and river system (mainstream vs. tributary), with SJ representing the Jialing River in spring, SX representing the Xichong River in spring, AJ representing the Jialing River in autumn, and AX representing the Xichong River in autumn. 

For the first time, multiple analytical methods including non-metric multidimensional scaling (NMDS), the neutral community model (NCM) combined with the modified stochasticity ratio (MST), and partial least squares path modeling (PLS-PM) were integrated and applied to the zooplankton community study in the Jialing River Basin. The objectives were to (1) determine the water ecological health status under different habitats (SJ, SX, AJ, AX) in the Jialing River Basin; (2) elucidate the assembly mechanisms of zooplankton communities in the basin; (3) by integrating the evaluation results of physicochemical water quality indices, diversity indices, the WQI, and the Z-IBI within a multi-model framework, preliminarily identify the potential associations between community assembly mechanisms and ecological health status, and explore their possible environmental driving pathways, thereby offering a scientific basis for water ecological health management and ecological restoration in the Jialing River Basin. Within the existing assessment framework, this study further explores the potential linkages between community assembly mechanisms and ecological health status. The findings will enhance our understanding of zooplankton communities and provide a theoretical foundation for basin-scale aquatic ecological conservation and the water ecological protection of urban rivers.

## 2. Materials and Methods

### 2.1. Study Area

The Jialing River originates from the Qinling Mountains, enters the Sichuan Basin, and traverses diverse climatic zones, landforms, and land use types. It is particularly sensitive to climate change and human activities, and ultimately joins the Yangtze River at Chaotianmen [23]. As the largest tributary of the Yangtze River in terms of drainage area and the second largest in terms of runoff (second only to the Minjiang River), its mainstream has a total length of 1345 km and a drainage area of 39,200 km^2^. The river’s runoff is primarily fed by rainfall, with discharge during the wet season (May–October) far exceeding that during the dry season (November–April), highlighting pronounced seasonal hydrological variability. Nanchong City is situated in a subtropical humid monsoon climate zone with four distinct seasons, characterized by a multi-year average temperature of approximately 17.5 °C and an average annual precipitation of about 980 mm, with rainfall concentrated from May to September [24,25,26,27]. Within Nanchong City, the Jialing River mainstream extends 301 km, flowing through three districts and four counties (cities), with an average annual discharge of 878.7 m^3^/s and an annual runoff volume of 27.76 billion m^3^. It serves as the primary source of centralized drinking water for Nanchong City [28]. As a first-order tributary on the west bank of the middle reaches of the Jialing River, the Xichong River has a total length of 121 km and a drainage area of 450 km^2^. It flows through 29 towns and 205 administrative villages, involving a population of 320,000. As a short-source, short-flow stream, the Xichong River exhibits significant seasonal variation in rainfall, with an annual total precipitation ranging from approximately 894.6 to 976.9 mm. Coupled with continuous inputs of industrial, agricultural, and domestic sewage along its banks, as well as limited flow and insufficient self-purification capacity, as a result, its aquatic ecosystem has shown a trend toward degradation [12].

### 2.2. Sampling Site Design

**Figure 1:**
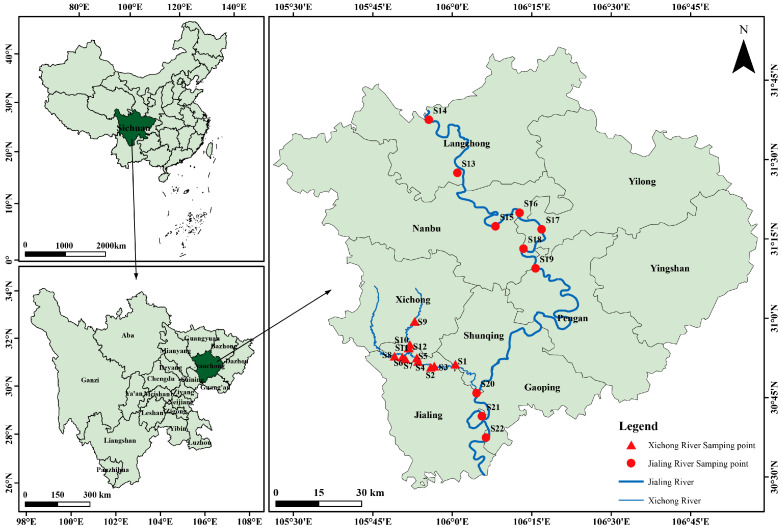
Sampling sites map of water ecological survey. Based on a systematic assessment of riparian land use types and potential disturbance sources through preliminary field reconnaissance, sampling sites in this study were established along an approximately 90–100 km section of the Jialing River mainstream, covering five representative disturbance types: natural river channel, engineering disturbance, tributary disturbance, sand mining disturbance, and agricultural reclamation disturbance. In the Xichong River tributary, sampling sites covered most of its course, encompassing four major representative disturbance types: natural river channel, urban disturbance, agricultural reclamation disturbance, and dammed river disturbance, which are considered representative of the typical anthropogenic pressures in the basin.

In this study, zooplankton samples were collected from the Jialing River Basin (mainstream) and the Xichong River Basin (tributary) within Nanchong City during October–November 2022 and March 2023. To minimize potential temporal interference with zooplankton community composition due to prolonged sampling intervals, all sampling sites within each season were completed within five consecutive days, and sampling periods avoided extreme rainfall or hydrological events. A total of 12 sampling sites (S1–12) were established in the Xichong River Basin. Based on the types of anthropogenic disturbances in the riparian zone, these sites were classified into four categories: natural river channel sites (S4, S8, S10), urban disturbance sites (S1, S9), agricultural reclamation disturbance sites (S3, S7, S11), and dammed river disturbance sites (S5, S6, S2, S12). In the Jialing River Basin, 10 sampling sites (S13–S22) were established and classified into five types: natural river channel sections (S15, S17), engineering disturbance sections (S14, S21), tributary disturbance sections (S19, S20), sand mining disturbance sections (S16, S18), and agricultural reclamation disturbance sections (S13, S22). The classification criteria were derived from field reconnaissance of all candidate sites prior to formal sampling, and the classification was validated by multiple experts (see Supplementary Materials Table S9 for details), with each disturbance type having 2–3 independent sections as spatial replicates. Based on seasonal and river type (mainstream vs. tributary) differences, the zooplankton samples were divided into four groups: Jialing River spring group (SJ), Jialing River autumn group (AJ), Xichong River spring group (SX), and Xichong River autumn group (AX). The layout of the study area and sampling points is shown in Figure 1.

### 2.3. Collection and Processing of Zooplankton Samples

According to the Technical Guidelines for Water Ecological Monitoring—River Aquatic Organism Monitoring and Assessment (HJ 1295—2023), water samples were collected from the upper, middle, and lower layers at each sampling site using a 5 L water sampler (totaling 15 L). After thorough mixing, a 1 L composite water sample was taken for zooplankton analysis. Immediately after field collection, samples were fixed on-site with Lugol’s iodine solution and transported to the laboratory under low-temperature and dark conditions. The samples were then concentrated by filtering through a 64 μm mesh plankton net and secondarily fixed with 4% formaldehyde solution. After standing for 48 h, the supernatant was removed by siphoning, and the concentrate was further condensed and adjusted to a final volume of 50 mL. The concentrated sample was thoroughly shaken, and an aliquot was placed into a 1 mL plankton counting chamber for enumeration [29,30]. Each sample was counted twice in parallel, and the average value was taken. The difference between each count and the mean was required to be no more than 15%; otherwise, an additional count was performed until two counts met this criterion. Specimens were identified based on morphology [31,32], and all zooplankton taxa were identified to the species level.

### 2.4. Determination of Physicochemical Parameters

The determination of water physicochemical parameters was divided into two parts: in situ measurement and laboratory analysis. In situ, key indicators including water temperature (WT), pH, conductivity (Cond), and dissolved oxygen (DO) were measured using a portable multi-parameter water quality analyzer. Water depth (WD) data were acquired with a handheld ultrasonic depth sounder, and water transparency (SD) was evaluated by the Secchi disk method. Laboratory analyses were performed according to the standard methods of China’s “Environmental Quality Standards for Surface Water” (GB 3838-2002, Supplementary Table S6) [33]. Total phosphorus (TP) was determined using the potassium persulfate digestion UV spectrophotometric method. Ammonia nitrogen (NH_3_-N) was measured via the Nessler’s reagent spectrophotometric method. The permanganate index (COD_Mn_) was determined using the acidic potassium permanganate titration method [12,34].

### 2.5. Data Processing and Analysis

ArcGIS Pro was used to generate the map of aquatic ecological sampling sites; Microsoft Excel and SPSS 27 were employed for data recording, processing, and analysis; Origin 2024 and RStudio (v4.5.1) were utilized to generate graphics from the collected, analyzed, and sorted data.

All significance tests were performed using SPSS 27 software. For comparisons of density, ***α*** diversity indices, and water quality parameters among the four groups (SJ, SX, AJ, AX), One-way ANOVA was employed for overall difference testing, followed by Tukey HSD post-hoc multiple comparisons for pairwise group differences. Prior to ANOVA, the homogeneity of variances across groups was verified. The significance level was set at ***α*** = 0.05. Rarefaction analysis was performed to assess sampling sufficiency and to support the robustness of subsequent community analyses (Figures S3 and S4).

In this study, ***α***-diversity analysis was conducted using the Shannon–Wiener index [35], the Pielou index [36], and the Margalef index [37].

$$H^{\prime }=-\overset{S}{\underset{i=1}{\sum }}\frac{n_{i}}{N}\log_{2}(\frac{n_{i}}{N})$$

where *H′* is the Shannon–Wiener diversity index; *N* is the total number of zooplankton individuals; *S* is the number of zooplankton species; *n_i_* is the number of individuals of the i-th zooplankton species.

$$D=\frac{\left(S-1\right)}{\ln N}$$

where *D* is the Margalef richness index; *N* is the total number of individuals; *S* is the total number of species.

$$J=\frac{H^{\prime }}{\ln S}$$

where *J* is the Pielou evenness index; *H′* is the Shannon–Wiener index; *S* is the number of species.

In this study, in situ parameters including WT, pH, Cond, DO, WD, and SD were only systematically measured at a subset of sampling sites, resulting in incomplete datasets that could not be incorporated into the WQI calculation. Consequently, only NH_3_-N, TP, and COD_Mn_ yielded complete and valid data across all sites. Therefore, these three water quality indicators—NH_3_-N (mg/L), TP (mg/L), and COD_Mn_ (mg/L)—were processed via range standardization [38,39], and the relative weight of each indicator was obtained by combined weighting using the CRITIC method and the entropy weight method (EWM) [40,41,42]. The final aggregated water quality index (WQI) was calculated to characterize water ecological health. The classification criteria are detailed in Table S1 of the Supplementary Materials [43].

$$W=\frac{\Sigma_{i=1}^{n}C_{i}P_{i}}{\Sigma_{i=1}^{n}P_{i}}$$

where *W* is the comprehensive water quality index (WQI); *n* is the total number of water quality parameters used to calculate the WQI; *C_i_* is the standardized factor of the i-th water quality indicator; and *P_i_* is the relative weight of the i-th water quality indicator.

In this study, the zooplankton Index of Biotic Integrity (Z-IBI) was used to assess river ecological health. Reference sites were determined based on the dual criteria of China’s Class II water quality standard and classification as natural river channels based on disturbance types [31]. Across the spring and autumn sampling seasons, a total of four reference sites were identified (SJ15, SJ17, AJ15, AJ17). Through range testing, discriminant ability analysis (retaining metrics with an interquartile range value ≥ 2 in boxplots; i.e., metrics for which the medians of both reference and disturbed sites fell outside each other’s box ranges, indicating good discriminant ability; see Figure S1), and Spearman correlation analysis (|r| < 0.75, see Figure S2), five core metrics—M4, M7, M12, M14, and M22—were selected from 22 candidate metrics (see Table S3). The 5th percentile (P5) and 95th percentile (P95) of each metric at reference sites were calculated. The ratio method was then applied to standardize the data: if the calculated ratio was less than 0, it was set to 0; if greater than 1, it was set to 1; the result was then multiplied by 100, so that the score of each metric ranged from 0 to 100 [44,45].

For positive indicators: standardized value = [(observed value − P5)/(P95 − P5)] × 100. For negative indicators: standardized value = [(P95 − observed value)/(P95 − P5)] × 100. For classification, the 95th percentile of Z-IBI values across all sampling sites was taken as the optimal reference value. Sites with Z-IBI values above this threshold were rated as “excellent”; the interval from 0 to the 95th percentile was equally divided into four segments, which were defined as “good”, “medium”, “poor”, and “very poor”, respectively (see Table S1).

All data analyses were conducted using RStudio. The following packages and methods were applied. Mantel tests were performed using the mantel_test() function from the linkET package to analyze the correlations between zooplankton density data and water quality parameters as well as diversity indices. The relative abundance data derived from density were used for NMDS ordination and neutral community model (NCM) analysis. The neutral community model was implemented using the Hmisc package: zooplankton relative abundance data were input to calculate species occurrence frequencies, and a non-linear least squares method was used to fit the frequency–relative abundance relationship to estimate the migration rate (Nm) and its confidence intervals(the 95% confidence interval for parameter m was calculated based on the profile likelihood method; the 95% confidence intervals for predicted frequencies were derived using the Wilson score interval method) [46,47,48,49]. The goodness-of-fit (R^2^) was obtained by predicting frequencies based on the fitted parameters, thereby quantifying the relative contributions of stochastic and deterministic processes to community assembly. The modified stochasticity ratio (MST) was calculated using the NST package. Partial least squares path modeling (PLS-PM) was performed using the pls-pm package to analyze the interactions among five latent constructs: water quality indicators, diversity indices, ecological assessment indices, community structure, and assembly mechanisms. All latent constructs were specified as reflective measurement models [19]. For the community structure construct, the observed indicators were the densities of Protozoa, Rotifera, Cladocera, and Copepoda. The observed indicators for the remaining four latent constructs are fully indicated in the subsequent figures and their corresponding notes.

## 3. Results and Analysis

### 3.1. Characteristics of Zooplankton Communities in the Jialing River Basin

#### 3.1.1. Spatial Variation in Relative Abundance Along the River and Spatiotemporal Changes in Density Under Different Disturbance Types

**Figure 2:**
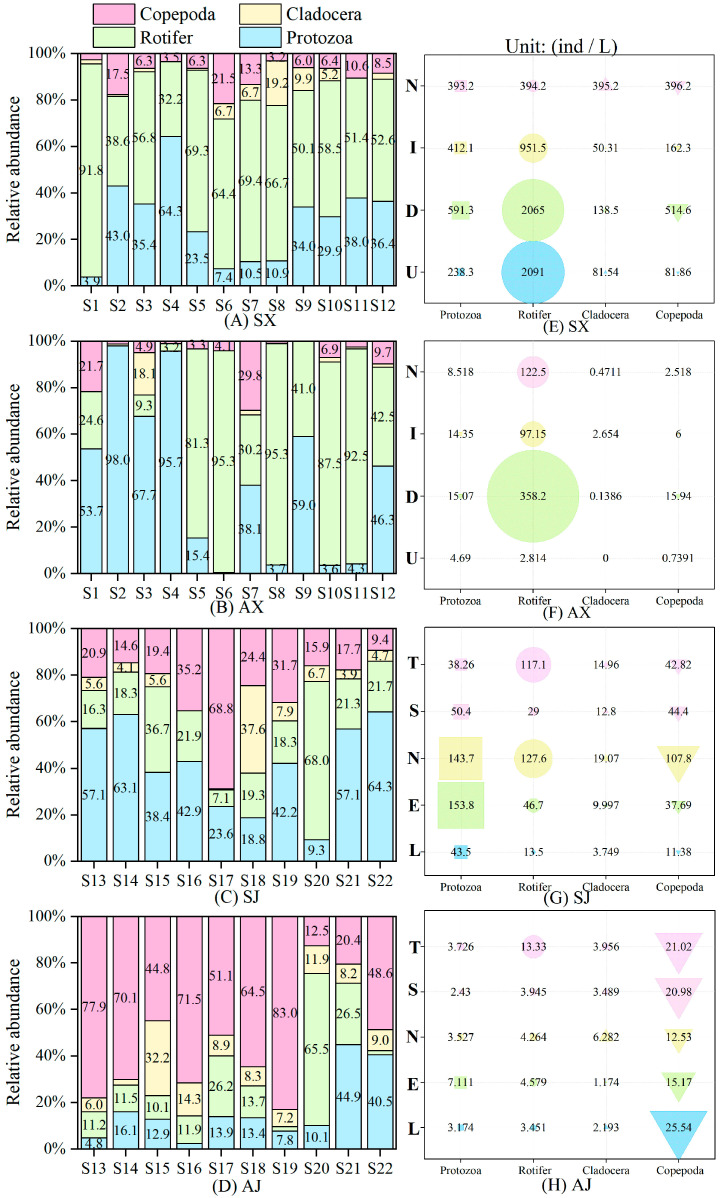
Group of diagrams showing temporal and spatial variations in the relative abundance and density of zooplankton communities. Subfigures (**A**–**D**) illustrate the longitudinal variations in community relative abundance; (**E**–**H**) show the density changes of various zooplankton species under different disturbances. In the figure, SX denotes spring in the Xichong River, AX denotes autumn in the Xichong River, SJ denotes spring in the Jialing River, and AJ denotes autumn in the Jialing River. U represents urban disturbance; D represents dam disturbance; L represents land reclamation disturbance; N represents natural river channel; E represents engineering disturbance; S represents sand mining disturbance; T represents tributary inflow disturbance. In this figure, the density and relative abundance of the four major zooplankton groups were aggregated based on species-level identification data of their constituent species. The complete species list and abundance data are provided in the Supplementary Materials (File S1).

As shown in Figure 2A–D, in the Xichong River during spring (SX), rotifers were the absolutely dominant group (mean relative abundance: 58.5%), whereas in autumn (AX), rotifers and protozoans were co-dominant (mean relative abundances: 50.3% and 40.6%, respectively). From spring to autumn, the mean relative abundance of copepods decreased from 8.8% to 7.2%, and that of cladocerans declined from 4.6% to 2.1%. In stark contrast, the zooplankton community structure in the Jialing River exhibited a clear succession, with copepod dominance increasing markedly in autumn: their mean relative abundance rose sharply from 25.8% in spring to 54.5%, whereas the mean abundance of protozoans dropped from 41.7% to 16.7%.

As shown in Figure 2E–H, the overall density distribution of zooplankton in the Jialing River Basin was significantly higher in spring than in autumn (Supplementary Table S2), and in spring, the density in the Xichong River was significantly higher than that in the Jialing River (*p* < 0.05; see Table S2 in the Supplementary Materials). For the Xichong River, at sites under human-disturbed habitats—specifically dam disturbance (D) and urban disturbance (U)—the community density exhibited pronounced changes, characterized by absolute dominance of rotifers, reflecting a drastic response of a single taxonomic group. The zooplankton density in the Jialing River was generally lower than that in the Xichong River. In spring, the lowest densities were observed at sites under cultivation disturbance (L) and natural channel (N), whereas sites under engineering disturbance (E) and sand mining disturbance (S) showed relative dominance of copepods. By autumn, copepods had become the clearly dominant group under sand mining disturbance (S) and tributary disturbance (T).

#### 3.1.2. Spatiotemporal Variation in Zooplankton Community Biomass

**Figure 3:**
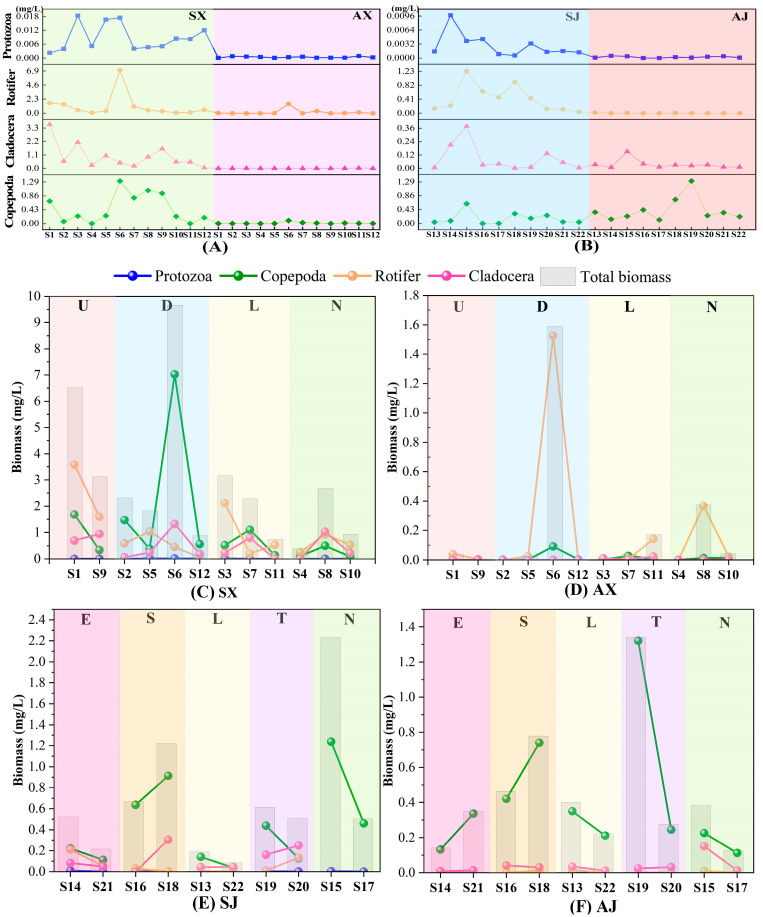
Variations in zooplankton community biomass. Subfigures (**A**,**B**) show the longitudinal biomass variations in the Xichong River and Jialing River basins. Subfigures (**C**–**F**) compare zooplankton biomass changes under different disturbances. In the figure, SX denotes spring in the Xichong River, AX denotes autumn in the Xichong River, SJ denotes spring in the Jialing River, and AJ denotes autumn in the Jialing River. U represents urban disturbance; D represents dam disturbance; L represents land reclamation disturbance; N represents natural river channel; E represents engineering disturbance; S represents sand mining disturbance; T represents tributary inflow disturbance. The biomass of each species was calculated based on species-level identification data, and the biomass of the four major groups was obtained by summing the values of their constituent species. Complete data are provided in the Supplementary Materials (File S1).

The zooplankton biomass in the Jialing River and Xichong River exhibited significant fluctuations between spring and autumn (shown in Figure 3A,B). Among them, the most dramatic change occurred in the Xichong River in spring, with the maximum range observed at site S6, reaching 8.46 mg/L and representing a 24.5-fold difference. In autumn, the total biomass decreased sharply, and the biomass across different taxonomic groups became relatively balanced.

Figure 3C–F shows the biomass values of different zooplankton groups under various disturbance types. At site S6 on the Xichong River under dam disturbance (D), explosive proliferation of a single taxonomic group was observed, with copepod biomass reaching as high as 7.03 mg/L, which was considerably higher than that at other sites. This anomaly may be related to local environmental changes induced by the dam and holds distinctive ecological significance. In the Jialing River during autumn, sand mining disturbance (S) and tributary disturbance (T) resulted in extremely high copepod biomass (S19: 1.32 mg/L; S18: 0.74 mg/L), significantly higher than that under cultivation disturbance and natural channel conditions. In spring, the maximum copepod biomass was observed at sand mining disturbance sites and natural channel sites, with site S15 (natural channel, N) showing the highest total biomass across both seasons.

#### 3.1.3. Correlation Analysis of Factors Influencing Different Zooplankton Groups

**Figure 4:**
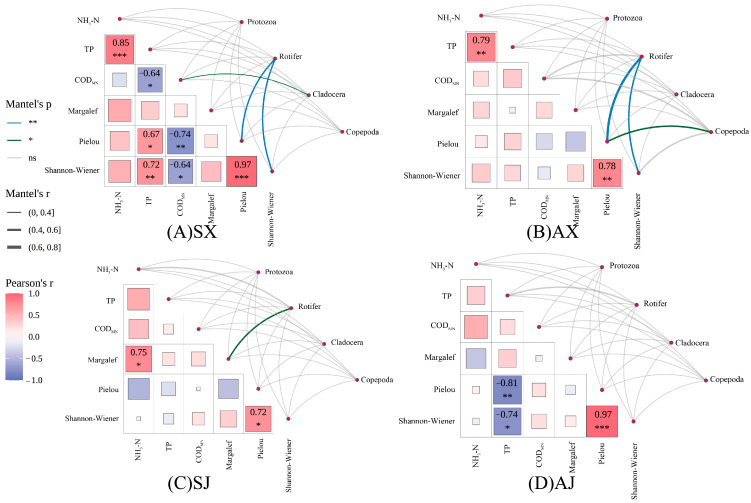
Correlations between zooplankton community structure and environmental factors and diversity indices (Mantel test and Pearson correlation). In the figure: (**A**) SX: spring in the Xichong River; (**B**) AX: autumn in the Xichong River; (**C**) SJ: spring in the Jialing River; (**D**) AJ: autumn in the Jialing River. For each panel, The lower-left triangular heatmap shows the Pearson correlation coefficients between environmental factors and diversity indices (color intensity indicates the strength of the correlation, with numerical values representing r). Mantel test was performed based on the Bray–Curtis dissimilarity matrix calculated from the raw zooplankton density data, while Pearson correlation analysis used the raw water quality parameter values and diversity index values. The upper-right section displays the Mantel test results, where line colors indicate significance levels (*** *p* < 0.001, ** *p* < 0.01, * *p* < 0.05), and line thickness represents the magnitude of Mantel’s *r*. The Pearson correlation coefficient (*r*) measures the strength and direction of the linear relationship between two environmental factors or diversity indices, ranging from −1 to 1.

Mantel tests were used to reveal the overall correlations between environmental factors and biological communities, while Pearson correlation analysis quantified the strength of linear relationships among environmental factors. The results are presented in Figure 4. As shown in Figure 4A, in the Xichong River during spring (SX), NH_3_-N and TP exhibited a very strong positive correlation (*r* = 0.85 ***), and both were negatively correlated with COD_Mn_. COD_Mn_ showed significant negative correlations with the Shannon–Wiener index (*r* = −0.64 *) and the Pielou index (*r* = −0.74 **), whereas TP showed significant positive correlations with the Shannon–Wiener index (*r* = 0.72 **) and the Pielou index (*r* = 0.67 *). In stark contrast, in the Jialing River during autumn (Figure 4D), TP exhibited significant negative correlations with the Shannon–Wiener index (*r* = −0.74 *) and the Pielou index (*r* = −0.81 **). We speculate that this discrepancy reflects a shift in the dominant stressor between the two river sections (water quality data are provided in Table 1 and Table S7): as a highly urbanized tributary, the Xichong River experiences continuous inputs from industrial, agricultural, and domestic sewage, resulting in significantly higher COD_Mn_ concentrations than those in the Jialing River mainstream, which in turn serves as a dominant factor limiting diversity. In contrast, the TP concentration in the Jialing River during autumn was markedly higher than that in spring and also higher than that in the Xichong River. TP thus becomes a key factor regulating phytoplankton growth and zooplankton food quality, potentially affecting zooplankton diversity indirectly through food web cascading effects.

As shown in Figure 4B, rotifers in the Xichong River were highly sensitive to diversity indices (Mantel’s *r* > 0.6), whereas the correlations between zooplankton groups and environmental factors in the Jialing River were relatively weaker. Overall, the strength of correlations among environmental factors was generally higher in the Xichong River than in the Jialing River. Furthermore, in the Xichong River, all four zooplankton groups—protozoans, cladocerans, copepods, and rotifers—consistently showed negative correlations with the two physicochemical indicators TP and NH_3_-N across all groups (Mantel’s *r* < 0), whereas rotifers in the Jialing River showed consistent positive correlations with TP and NH_3_-N (Mantel’s *r* > 0) (Detailed numerical results are provided in Table S5).

### 3.2. Alpha Diversity Index Analysis of the Jialing River Basin

**Figure 5:**
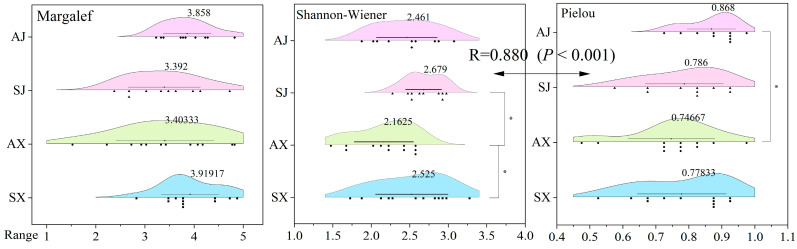
***α*** diversity analysis of zooplankton communities. The raincloud plots combine a half-violin plot (cloud) showing the probability density distribution, a boxplot (black lines) displaying the median and interquartile range, and jittered raw data points (dots) representing individual sampling site values. *R* represents the correlation coefficient. In the figure, SX denotes the Xichong River in spring, AX denotes the Xichong River in autumn, SJ denotes the Jialing River in spring, and AJ denotes the Jialing River in autumn. The Shannon–Wiener index, Pielou index, and Margalef index were all calculated based on raw species-level data (identified to species level), without data transformation or standardization. The results of one-way ANOVA are provided in Supplementary Material Table S8. Significance levels are indicated by asterisks: * *p* < 0.05.

The correlation analysis (Figure 5) indicates a highly significant positive correlation between the Shannon–Wiener index and the Pielou index in the Jialing River Basin (*R* = 0.880, *p* < 0.001). In terms of the Margalef index, the highest value was observed in the Xichong River in spring (SX: 3.919), and the lowest in the Jialing River in spring (SJ: 3.392). The Shannon–Wiener index was highest in the Jialing River in spring (SJ: 2.679) and lowest in the Xichong River in autumn (AX: 2.1625). The Pielou index was highest in the Jialing River in autumn (AJ: 0.868) and lowest in the Xichong River in autumn (AX: 0.747). One-way ANOVA showed that significant differences were found in the Shannon–Wiener index between the Xichong River in autumn (AX) and the Xichong River in spring (SX), as well as between AX and the Jialing River in spring (SJ). Significant differences were also found in the Pielou index between the Xichong River in autumn (AX) and the Jialing River in autumn (AJ). However, no significant differences in the Margalef index were observed among the four groups, suggesting that the sensitivity of this index to community changes may be lower than that of the Shannon–Wiener and Pielou indices. Complete statistical test results for each diversity index are provided in Supplementary Materials Table S8. Overall, the ***α*** diversity indices of zooplankton communities were higher in autumn than in spring, and higher in the mainstream than in the tributary.

### 3.3. NMDS Analysis Based on Zooplankton Communities

**Figure 6:**
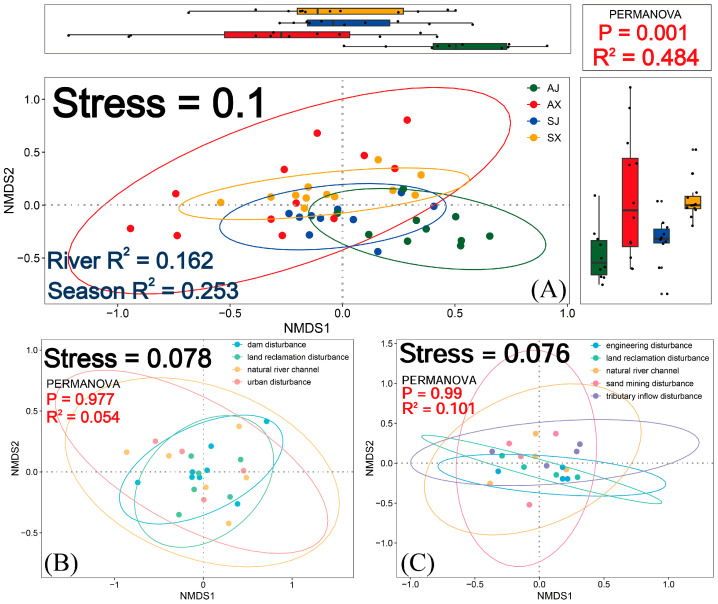
Non-metric multidimensional scaling (NMDS) ordination of zooplankton communities in the Jialing River Basin. The NMDS ordination was based on the Bray–Curtis dissimilarity matrix calculated from the raw zooplankton density data. The Stress value indicates the goodness-of-fit of the ordination; generally, Stress < 0.1 is considered a good fit, rendering the plot interpretable. Each point represents a sampling site; the closer the points, the more similar the community composition. Panel (**A**) shows samples grouped by the combination of season and mainstream/tributary status. Panel (**B**) shows samples grouped by disturbance type in the Xichong River. Panel (**C**) shows samples grouped by disturbance type in the Jialing River. The *R*^2^ and *p* values shown in the figure are derived from PERMANOVA tests, where *R*^2^ represents the proportion of total community variation explained by the grouping factor, and *p* < 0.05 indicates a significant grouping effect. In Panel (**A**), *R*^2^ = 0.162 is from a one-factor PERMANOVA with “mainstream vs. tributary” as the sole grouping factor; *R*^2^ = 0.253 is from a one-factor PERMANOVA with “season” as the sole grouping factor; and *R*^2^ = 0.484 is from a one-factor PERMANOVA with the “combination of season and mainstream/tributary” (i.e., SJ, SX, AJ, AX) as the sole grouping factor. In the figure, SJ denotes the Jialing River in spring, AJ denotes the Jialing River in autumn, SX denotes the Xichong River in spring, and AX denotes the Xichong River in autumn.

As shown in Figure 6A, when samples were grouped by mainstream versus tributary (Stress = 0.12, *R*^2^ = 0.162, *p* = 0.001), PERMANOVA explained 16.2% of the community variation. When grouped by season (Stress = 0.09, *R*^2^ = 0.253, *p* = 0.001), the analysis explained 25.3% of the community variation. This indicated that among all grouping factors in this study, seasonal change was the primary factor influencing zooplankton community composition. Grouping by both season and river type together explained 48.4% of the community variation (Stress = 0.1, *R*^2^ = 0.484, *p* = 0.001), suggesting that the combination of season and mainstream/tributary may provide additional explanatory power for community structure beyond additive effects. For the Xichong River (Figure 4B, Stress = 0.078), points representing different disturbance types were highly overlapping, and the PERMANOVA result was not significant (*R*^2^ = 0.054, *p* = 0.977). Similarly, for the Jialing River (Figure 4C, Stress = 0.076), no effective separation among disturbance types was observed (*R*^2^ = 0.101, *p* = 0.99). This may imply that the key factor determining community response is not the disturbance type per se, but rather requires consideration of quantitative measures of disturbance intensity or the actual degree of habitat condition alteration.

### 3.4. Integrated Index Evaluation of the Jialing River Basin

#### 3.4.1. Evaluation of Water Quality Characteristics in the Jialing River Basin

As shown in Table 1, the three physicochemical indicators—TP, NH_3_-N, and COD_Mn_—were overall significantly higher in the Xichong River than in the Jialing River. Moreover, TP and NH_3_-N in the Xichong River also showed significant differences between spring and autumn, whereas the Jialing River maintained relatively good water quality throughout the year with no significant seasonal differences. According to the comprehensive water quality index (WQI), the overall water quality of the basin was better in autumn than in spring. Specifically, the mainstream of the Jialing River exhibited good water quality, with the best conditions occurring in autumn(79.26). In contrast, the Xichong River was rated as “poor” in both spring and autumn, with the worst conditions in autumn(34.34); its mean concentrations of NH_3_-N, TP, and COD_Mn_ reached 0.873 mg/L, 0.128 mg/L, and 6.841 mg/L, respectively, far exceeding the physicochemical parameters observed in other habitats (detailed site-specific data are provided in Supplementary Materials Table S7).

#### 3.4.2. Analysis of the Zooplankton Index of Biotic Integrity (Z-IBI)

Based on discriminant ability analysis and Spearman correlation analysis, five core metrics were selected from 22 candidate metrics: Number of Copepoda species (M4), Rotifer density (M7), Percentage of Rotifer density (M12), Percentage of Copepoda density (M14), and Shannon–Wiener index (M22). All candidate metrics are listed in Table S3. The Z-IBI scores were ranked as follows: AJ (65.60) > SJ (52.95) > AX (26.89) > SX (17.83). The Jialing River in autumn achieved the highest Z-IBI score, with an overall rating of “good”; the Jialing River in spring ranked second with a score of 52.95, rated as “medium”; the Xichong River in autumn followed with an average Z-IBI of 26.89, rated as “poor”. Spatially, the average Z-IBI of the Jialing River (59.28) was significantly higher than that of the Xichong River (22.36). Temporally, the average Z-IBI of the entire basin in autumn (44.48) was significantly higher than that in spring (33.80).

In summary, the Z-IBI assessment results were consistent with the WQI-based water quality assessment at the basin scale: the mainstream was better than the tributary, and autumn was better than spring. However, in the highly urbanized Xichong River, the two assessment indices showed asynchrony. (Detailed site-specific assessment results are provided in Table S4).

### 3.5. Analysis of Zooplankton Community Assembly Mechanisms and Driving Pathways in the Jialing River Basin

**Figure 7:**
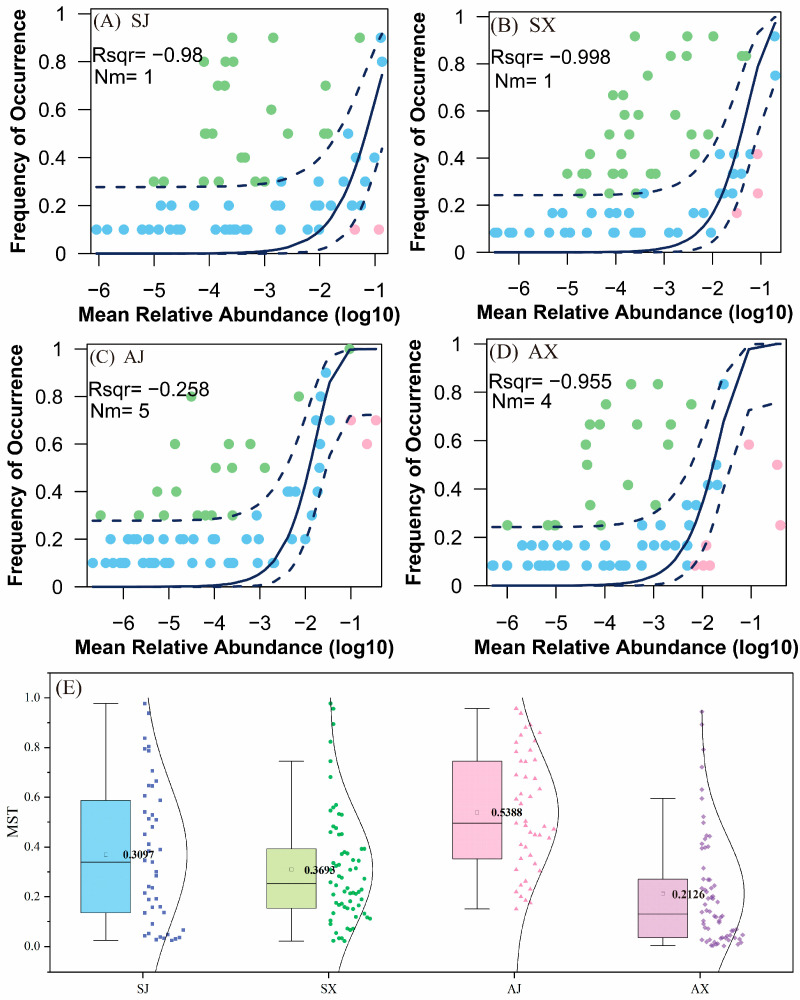
Neutral model analysis based on zooplankton community. Figures (**A**–**D**) present the results from the Neutral Community Model; Figure (**E**) shows the results derived from MST values. The NCM analysis was based on the relative abundance of the four major zooplankton groups, while the MST analysis was based on density data. In Figures (**A**–**D**), the solid blue line represents the best-fit curve, while the dashed blue lines indicate the upper and lower 95% confidence intervals, respectively. *R*^2^ denotes the goodness of fit, where a higher goodness of fit suggests a greater influence of stochastic processes on zooplankton community assembly; Nm represents migration rate, with higher Nm values indicating a stronger influence of deterministic processes on zooplankton community assembly. In this study, most groups in the Neutral Community Model (NCM) exhibited significantly negative *R*^2^ values, strongly rejecting the qualitative diagnosis of the neutral hypothesis (dominance of stochastic processes) and indicating that deterministic processes (environmental filtering) play a dominant role. Building on this, the MST model offers a standardized quantitative framework, where an MST value less than 0.5 indicates that deterministic processes dominate community assembly, while a value greater than 0.5 suggests dominance by stochastic processes. Thus, the negative *R*^2^ values from the NCM model reveal the direction and extent of deviation from stochastic mechanisms, while the MST translates qualitative descriptions into comparable quantitative indicators. The two approaches describe community assembly mechanisms from different dimensions, with the MST index serving as the primary basis for quantitative judgment.

The neutral community model (NCM) results, shown in Figure 7A–D, indicated that the *R*^2^ values for all four groups in the Jialing River Basin were significantly negative, strongly rejecting the neutral hypothesis at the qualitative diagnostic level. This suggests that zooplankton community assembly in the basin as a whole was not dominated by stochastic processes. To more comprehensively describe the community assembly mechanisms, we combined this with the quantitative analysis using the MST index (Figure 7E). Only the zooplankton community in the Jialing River during autumn (AJ) was dominated by stochastic processes (MST = 0.5388); however, the NCM fit *R*^2^ for this group was only −0.258 (Nm = 5), indicating that this stochasticity was not driven by classical neutral processes. The remaining three groups (SJ, SX, AX) all exhibited clear deterministic dominance (MST < 0.5) under the MST evaluation, which was consistent in trend with the NCM results (all *R*^2^ values were negative): the Xichong River in spring (SX, *R*^2^ = −0.998), the Jialing River in spring (SJ, *R*^2^ = −0.98), and the Xichong River in autumn (AX, *R*^2^ = −0.955).

**Figure 8:**
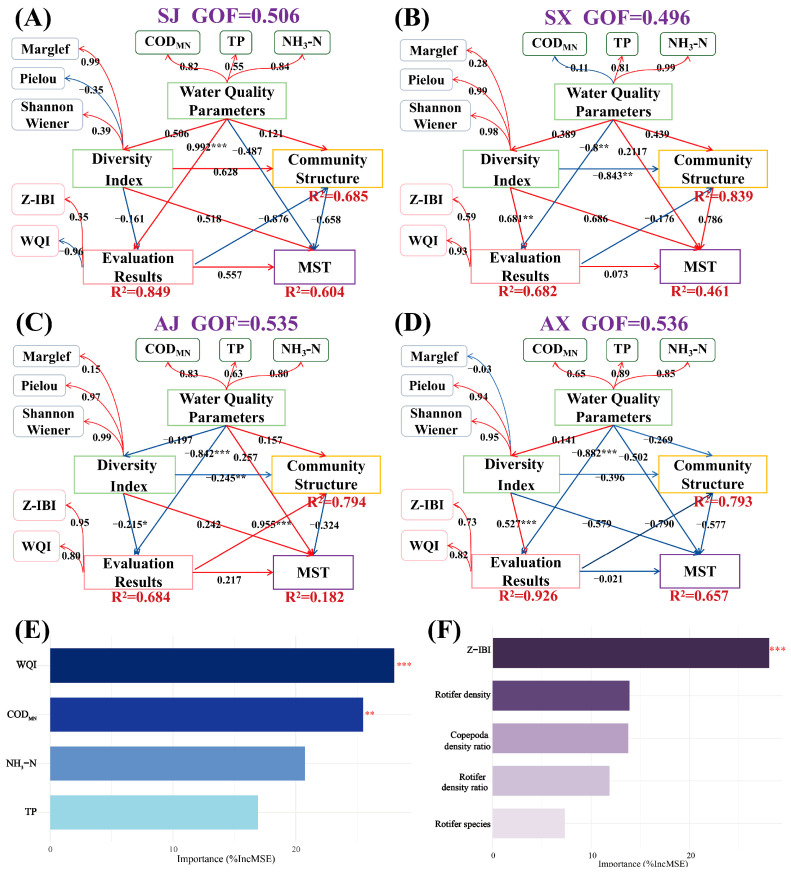
PLS-PM structural equation modeling and random forest importance ranking. Panels (**A**–**D**) show the path diagrams derived from PLS structural equation modeling. Unidirectional arrows indicate the hypothesized direction of influence, and the standardized path coefficients along the arrows represent the magnitude and direction of the effect (positive values indicate promotion, negative values indicate inhibition). *R*^2^ indicates the percentage of variance in a latent variable that can be explained by other latent variables pointing to it; generally, *R*^2^ > 0.2 is considered to indicate a certain explanatory power. The overall goodness-of-fit (GOF) of the model is used to evaluate model quality, and GOF > 0.36 is generally considered acceptable. All latent constructs were specified as reflective measurement models. The observed indicators corresponding to each latent construct were as follows: three water quality parameters (NH_3_-N, TP, COD_Mn_), three α diversity indices (Shannon–Wiener index, Pielou index, Margalef index), two water ecological assessment results (WQI, Z-IBI), four zooplankton community structure indicators (densities of Protozoa, Rotifera, Cladocera, and Copepoda), and the MST (modified stochasticity ratio) value. All observed indicators used raw data (density, index values, WQI and Z-IBI scores), with standardization completed through iterative weight calculations within the algorithm. The random forest importance ranking was calculated using the MST index and the raw values of each candidate indicator. Panels (**E**,**F**) show the random forest importance ranking of variables with respect to the MST index. The %IncMSE value represents the percentage increase in the mean squared error of model prediction when the variable is randomly permuted. The higher the value, the stronger the explanatory power of the variable for the MST index. All 22 candidate indicators related to the zooplankton community were included as independent variables in the importance ranking to identify the community structure indicators that contributed most to the MST. Significance levels are indicated by asterisks: *** *p* < 0.001, ** *p* < 0.01, * *p* < 0.05.

To further dissect the assembly mechanisms of zooplankton communities, this study employed partial least squares path modeling (PLS-PM) to perform path analysis on five latent variables: water quality physicochemical indicators, biodiversity indices, ecological assessment indices, zooplankton community structure, and community assembly mechanisms (MST). As shown in Figure 8A–D, the goodness-of-fit (GOF) values for the four groups ranged from 0.496 to 0.536, all exceeding 0.36, indicating moderate to good explanatory power. The variance explained (R^2^) of the comprehensive assessment results, zooplankton community structure, and community assembly mechanisms (MST) were as follows: SJ (R^2^ = 0.849, 0.685, 0.604), AJ (R^2^ = 0.684, 0.794, 0.182), SX (R^2^ = 0.682, 0.839, 0.461), and AX (R^2^ = 0.926, 0.793, 0.657). The path from water quality physicochemical parameters to the assessment results was significant across all groups (SJ = 0.902 ***, AJ = 0.842 *, SX = 0.8 **, AX = 0.882 ***). The path from diversity indices to the assessment results was significant in most groups (AJ = 0.215 *, SX = 0.681 **, AX = 0.527 ***). Moreover, the path coefficient from assessment results to zooplankton community structure was relatively high in most groups (SJ = 0.876, AJ = 0.955 ***, AX = 0.790).

The MST index showed a strong correlation with community structure (path coefficients: SJ = 0.658, SX = 0.786, AX = 0.577). Further combined with the random forest importance ranking results (Figure 8E,F), both the WQI and Z-IBI had significant effects on MST. According to the importance ranking of water quality indicators, the MST index was most sensitive to COD_Mn_ (importance = 25.48), followed by NH_3_-N (importance = 20.74), and then TP (importance = 16.92). Based on the zooplankton community analysis, the 22 candidate IBI metrics were ranked by importance with respect to the MST index, and rotifer density, proportion of copepods, proportion of rotifers, and rotifer species richness were identified as the four most important metrics.

## 4. Discussion

### 4.1. Analysis of Spatiotemporal Heterogeneity of Zooplankton Communities and Its Environmental Drivers

Changes in water temperature, hydrology, and nutrient dynamics induced by habitat alteration, together with spatial heterogeneity and anthropogenic stressors, collectively constitute the key driving forces shaping zooplankton community structure [50]. As intermediate links in the aquatic food chain, zooplankton are sensitive indicators of changing environmental conditions [1]. The present study revealed that zooplankton communities in the Jialing River Basin exhibited pronounced seasonal differences. Specifically, the density and biomass of zooplankton in the basin as a whole were significantly higher in spring than in autumn. Based on non-metric multidimensional scaling (NMDS) and PERMANOVA, seasonal succession was identified as the primary driver regulating changes in zooplankton community structure in the Jialing River Basin (Stress = 0.09, *R*^2^ = 0.253), explaining 25.3% of the community variation. This explanatory power was notably higher than that of spatial differences between the mainstream and tributaries (*R*^2^ = 0.162), echoing the findings of Bian et al. (2025) [51] on plankton community structure in the Yangtze River Basin (which focused on urbanized river sections such as Wuhan and Nanjing, covering seasonal comparisons between dry and wet seasons). These results indicate that seasonal variation periodically modulates the physical, chemical, and biological properties of water bodies [52], imposing a strong ecological filtering effect on biological communities.

Further Pearson correlation analysis revealed that in the AJ, AX, and SJ habitats, NH_3_-N, TP, and COD_Mn_ were all positively correlated with each other in pairs. Notably, in the Xichong River during spring (SX), NH_3_-N and TP exhibited a very strong positive correlation (r = 0.85, *p* < 0.001), suggesting that nitrogen-phosphorus nutrients and organic compounds may share common sources, such as agricultural non-point source pollution or urban domestic sewage.

Combined with Mantel test results, all zooplankton groups in the Xichong River exhibited negative correlations with TP and NH_3_-N in both spring and autumn (Mantel’s *r* < 0; see Table S5). Combined with our field surveys and historical data, the average concentrations of TP, NH_3_-N, and COD_Mn_ in the Xichong River were indeed higher than those in the Jialing River mainstream, indicating sustained environmental stress. From a spatial perspective, both the Jialing River and the Xichong River exhibited substantial proliferation of a few dominant species or a single taxonomic group at certain sites, with significantly higher density and biomass than at other sites, and the magnitude of fluctuation was also greater in spring than in autumn. This is not a data error, but rather a genuine ecological response of the ecosystem under disturbance. Taking the anomalously high copepod biomass at site S6 in the Xichong River during spring (7.03 mg/L) as an example, this may be attributed to the dam, which reduces flow velocity and prolongs hydraulic retention time in the upstream section, leading to the retention and aggregation of large filter-feeding zooplankton. Additionally, the area upstream of the dam tends to accumulate nutrients, promoting phytoplankton growth and thereby providing ample food for copepods. Zhang et al. (2025) [53] suggested that such simplification and imbalance of community structure are typical indicators of declining ecosystem resilience. This may imply that when disturbances reach a certain threshold, they create “more competitive” ecological conditions for specific taxonomic groups by altering flow conditions, substrate stability, or food web structure [54].

Studies have shown that the accumulation of nutrients and organic matter can promote plankton proliferation [1]. Concurrent research results indicated that [12]: in the Jialing River mainstream, phytoplankton were dominated by diatoms and green algae, with relatively high biodiversity; whereas in the Xichong River tributary, high nutrient loads led to a simplified phytoplankton community structure, with cyanobacteria and green algae being dominant, characterized by high biomass but low diversity. As the primary food source for zooplankton, changes in phytoplankton community structure can directly affect the food resources available to zooplankton [55,56]. The above phytoplankton patterns are largely consistent with the zooplankton responses observed in this study: under high nutrient loads in the Xichong River, zooplankton density and biomass surged, with more pollution-tolerant rotifers being selected as the dominant group [29]. In contrast, the seasonal succession of the zooplankton community in the Jialing River (protozoans dominant in spring, copepods dominant in autumn) more closely aligns with the ecological characteristics of a healthy river, which is similar to the findings of Wang et al. (2024) [28] on zooplankton in Jiaozhou Bay. It should be noted that, due to project constraints, phytoplankton-related indicators (such as Chl-a concentration) were not simultaneously measured, preventing direct quantification of their contribution to zooplankton. In future studies, we will incorporate quantitative phytoplankton indicators and community composition analyses, which we believe will help more comprehensively reveal the underlying driving mechanisms.

### 4.2. Comprehensive Water Ecological Assessment of the Jialing River Basin

This study conducted a comprehensive water ecological assessment of the Nanchong section of the Jialing River Basin based on two different evaluation indices: WQI and Z-IBI. The assessment results for water quality indices ranked as follows: autumn mainstream (WQI = 79.78) > spring mainstream (WQI = 79.26) > spring tributary (WQI = 49.22) > autumn tributary (WQI = 34.34), whereas the biotic integrity index results ranked as: autumn mainstream (Z-IBI = 65.60) > spring mainstream (Z-IBI = 52.95) > autumn tributary (Z-IBI = 26.89) > spring tributary (Z-IBI = 17.83). Under the combined evaluation of both indices, the mainstream (Jialing River) maintained an overall rating of “good”, whereas its tributary (Xichong River) maintained an overall rating of “poor”. Notably, seasonal variation led to asynchrony between the IBI and WQI evaluations in the Xichong River, which is consistent with the findings of Yongo et al. (2023) [57] for three urban rivers in Haikou City.

According to the water quality parameter measurements (Table 1), we found that the concentrations of TP, NH_3_-N, and COD_Mn_ in this river were higher in autumn, resulting in a lower WQI in the autumn tributary than in the spring tributary. In contrast, the Z-IBI evaluation showed the opposite trend: the spring tributary scored lower than the autumn tributary. The IBI is a comprehensive index synthesized from multiple metrics of biological communities, and studies have demonstrated that the IBI exhibits higher sensitivity than the WQI in ecological assessment [57,58]. Furthermore, Yan et al. (2025) [14] argued that physicochemical parameters merely reflect instantaneous water quality and that such assessments suffer from a time lag, whereas biological communities integrate historical environmental pressures, enabling a more complete judgment of ecosystem health and being more suitable for application in water ecological assessments of urbanized rivers.

Based on the Z-IBI analysis, the proportions of sites rated as “excellent”, “good”, and “medium” in the Jialing River during spring and autumn together accounted for 85%, with only one site approaching the “very poor” threshold (IBI < 25) (SJ20, Z-IBI = 27.86). In contrast, for the Xichong River, the proportions of sites rated as “very poor” and “poor” reached as high as 75%, and all remaining sites were rated as “Medium”, indicating a pronounced state of degradation. Oliver et al. (2015) [59] suggested that degraded ecosystems themselves possess higher uncertainty and vulnerability, making their assessment results more prone to fluctuations depending on the method used. As a typical tributary of the Jialing River Basin (Nanchong section), the Xichong River passes through 29 towns and 205 administrative villages, exhibiting a high degree of urbanization [12]. This further explains the discrepancy between the IBI-based and WQI-based evaluation results.

Notably, although the overall water ecological condition of the Xichong River is severely impaired, six sites still achieved a “medium” rating in the Z-IBI assessment (AX2, AX3, AX4, AX7, SX10, SX12). These sites all avoided urban disturbance types and were distributed in river sections with relatively high natural vegetation coverage and low human impact. By analyzing the WQI values at these sites, we found that the WQI values were not distinctly different from those at sites rated as “Poor” or “Very Poor,” indicating that the occurrence of the “medium” rating was not directly determined by differences in individual water quality indicators alone. This phenomenon may suggest that even when water quality indicators show no significant improvement, local habitats with relatively stable hydrological conditions, higher natural vegetation coverage, or greater habitat complexity may still maintain a certain degree of biological integrity, thereby retaining potential ecological self-regulation capacity [60]. Meanwhile, the NMDS grouping tests based on anthropogenic disturbance types showed that zooplankton communities did not exhibit significant differentiation (Figure 6B: Xichong River, *R*^2^ = 0.054; Figure 6C: Jialing River, *R*^2^ = 0.101). This result further suggests that the key factor determining community response may not depend entirely on the “disturbance type” per se, but rather on whether the disturbance intensity exceeds a certain ecological threshold, i.e., the actual degree of habitat condition alteration. Of course, this speculation is limited by the fact that we did not systematically quantify local environmental factors such as hydrological conditions and habitat complexity in this study, and thus requires further verification. Nevertheless, this finding also provides a new entry point for our future research: for urbanized rivers, developing scientifically sound management approaches should not only focus on highly sensitive, highly polluted areas, but also pay greater attention to regions with relatively better ecological status, exploring the mechanisms that maintain their relative health, thereby providing key references for the protection, restoration, and management of such impaired rivers.

### 4.3. Quantifying Zooplankton Community Assembly Mechanisms and Identifying Dominant Pathways in the Jialing River Basin Using Multi-Model Approaches

Freshwater ecosystems harbor extremely rich, unique, and sensitive biological communities, and their ecosystem services are modulated by changes in biodiversity, ecological processes, and spatial, temporal, and environmental characteristics [50,61]. In recent years, we have come to recognize that the recovery of aquatic organisms is central to rebuilding the structural and functional stability of river ecosystems [60]. As direct endpoints of environmental stress, biological communities can intuitively reflect environmental health status [62]. Zooplankton, as key bioindicators of ecosystem structure and function, can effectively reflect water ecological health through their community characteristics [1]. However, current mechanistic explorations of water ecology based on zooplankton remain insufficient [63]. How to accurately quantify changes in zooplankton community structure and establish linkages with environmental factors remains a fundamental challenge [64].

Accordingly, this study quantified the assembly mechanisms of zooplankton communities in the Jialing River Basin using the neutral community model (NCM) combined with the modified stochasticity ratio (MST). The NCM results strongly rejected the neutral hypothesis at the qualitative level [16,65], indicating that deterministic processes dominated community assembly. The MST index uses a threshold of 50% to distinguish between stochastic (MST > 0.5) and deterministic (MST ≤ 0.5) dominance [17]. The results showed that the MST values for the Jialing River in spring (SJ), the Xichong River in spring (SX), and the Xichong River in autumn (AX) were all below 0.5, indicating deterministic dominance, which is consistent with the NCM results. In contrast, the MST value for the Jialing River in autumn (AJ) was slightly above 0.5 (0.5388), indicating stochastic dominance. Given that the NCM fit R^2^ for this group was −0.258 (Nm = 5), this stochasticity likely originated from external random disturbances such as hydrological fluctuations and anthropogenic perturbations in the Jialing River [21], rather than from neutral processes based on species equivalence. From an ecological mechanism perspective, deterministic dominance implies that community structure is primarily shaped by non-random factors such as environmental filtering and interspecific competition. For example, in the Xichong River under high pollution stress, environmental filtering is sustained, leading to community homogenization (e.g., the absolute dominance of rotifers). In contrast, the enhanced stochasticity in the Jialing River during autumn may reflect that, under conditions of relatively low organic pollution and good water quality, stochastic factors gain more room to operate, resulting in a more diversified community composition.

Combined with the previous water ecological assessment results, the Jialing River in autumn (AJ) represented the period with the best water ecological health status in the basin, whereas the Xichong River in spring (SX) represented the group with the most severe water ecological degradation. Linking the MST results with the water ecological assessment outcomes may help explore the potential relationship between community assembly mechanisms and ecosystem health status. Further integrating the findings of Hu et al. (2024), Zeng et al. (2026), and Zhao et al. (2021) [7,20,66] on plankton communities, we hypothesize that in the Jialing River Basin, there is a close relationship between zooplankton community assembly mechanisms and ecosystem health status. Specifically, good water ecological conditions may be associated with stochastic processes dominating zooplankton communities, whereas deterministic dominance may indicate ecological degradation or a state of disturbance. It should be noted that this inference is based solely on data from four groups across two seasons (spring and autumn). We fully recognize that, given the limited sample size and temporal scale, it is insufficient to establish a universal ecological pattern. In future studies, we will increase sampling frequency and the number of sites to validate the applicability of this pattern across different spatiotemporal scales.

To validate the above hypothesis, a specific path analysis was conducted using the PLS-PM model. As shown in Figure 7, water quality status is an important exogenous driver of ecological responses, and changes in biodiversity are closely related to ecological health status. In relevant studies, water quality parameters can affect zooplankton communities through two pathways: a direct pathway—high concentrations of organic pollutants reduce dissolved oxygen levels, exerting physiological stress on zooplankton and directly inhibiting the survival and reproduction of sensitive groups (such as cladocerans and large copepods) [62,67]; and an indirect pathway—nutrient enrichment alters phytoplankton community structure [12], which in turn affects zooplankton food resources through food web cascading effects [56], ultimately manifesting as a simplification of community composition toward pollution-tolerant groups (rotifers). To further identify the key factors influencing community assembly mechanisms, this study employed random forest modeling to rank the importance of factors affecting the MST index (Figure 8E,F). The results showed that among the water quality indicators, the MST index was most sensitive to COD_Mn_ (importance = 25.48), followed by NH_3_-N and TP, suggesting that organic pollution is an important environmental filtering force driving community assembly, followed by nutrient loading. Among zooplankton groups, copepods and rotifers were identified as the two most important contributors, which is consistent with the community structural changes described in the first part of the discussion. Rotifers, with their short generation time and high reproductive capacity, can respond rapidly and become dominant under high pollution stress [68,69]; whereas copepods, with their longer life cycles and more demanding environmental requirements, typically indicate improved habitat conditions and more complex food web structures [70]. The fact that these two groups ranked as the top two contributors in the MST importance ranking further confirms the selective role of environmental filtering on functional traits during community assembly.

In summary, we obtained a relatively clear and verifiable causal pathway: seasonal variation as the dominant driving factor induced changes in water quality physicochemical parameters and biodiversity, which jointly altered zooplankton community structure, leading to differentiated ecological assessment results across the Jialing River Basin. Moreover, the combined application of the NCM (MST), RF, and PLS-PM models successfully captured the structural changes in zooplankton communities, ultimately manifesting as a quantifiable community assembly mechanism. Thus, the integrated WQI and Z-IBI assessment approach and the multi-model coupling quantitative framework employed in this study, preliminarily outline the potential linkages of “external stress input → biological community response → ecological process reconstruction”, providing a methodological reference for complex urban watersheds characterized by multiple pollution sources, hydrological disturbances, and high spatial heterogeneity [71,72]. It should be noted that to obtain a clearer causal pathway, further validation through more time-series data and experimental verification is required. In addition, environmental factors not included in this study, such as hydrological flow, hydrodynamic parameters, as well as pesticides and heavy metals, may influence community structure through multiple pathways. In our future research, we will prioritize the integration of these factors into a comprehensive analytical framework to establish a more complete and robust causal inference pathway.

Based on the above findings, water ecological management strategies should shift from “homogenized governance” to “spatiotemporal dynamic regulation” grounded in ecological processes. In spring, the entire basin is dominated by strong deterministic processes, with environmental filtering serving as the core driving force of community restructuring. Accordingly, management should focus on source control of key environmental factors, particularly in tributaries such as the Xichong River, where excessive inputs of nutrients and organic pollutants must be strictly controlled to mitigate community degradation induced by eutrophication. In autumn, community assembly mechanisms diverge: the mainstream shifts to stochastic process dominance, and management priorities should therefore turn to maintaining hydrological connectivity and habitat integrity to ensure natural succession and dispersal of biological communities. Furthermore, it is recommended to establish an early warning system alongside routine monitoring, and to conduct regular biological sampling to enable timely pollution source tracking and ecological regulation. This classification-based guidance, rooted in seasonal differences in community assembly mechanisms and the distinct characteristics of mainstream and tributary reaches, can more precisely enhance the stability and restoration efficiency of the Jialing River Basin ecosystem.

## 5. Conclusions

1. The spatiotemporal variation in zooplankton community biomass and density in the Jialing River Basin exhibited the following patterns: values were higher in spring than in autumn, and higher in the tributary than in the mainstream. Copepods dominated the mainstream, whereas rotifers prevailed in the tributary, indicating significant spatiotemporal heterogeneity in zooplankton community structure in the Jialing River Basin.
2. The overall water ecological status of the Jialing River Basin was better in autumn than in spring, and better in the mainstream than in the tributary. The assessment results ranked as: autumn mainstream > spring mainstream > autumn tributary > spring tributary. Seasonal variation played an important role in shaping zooplankton community assembly.
3. Seasonal changes in water quality drive community assembly mechanisms by altering zooplankton community composition. Among the physicochemical parameters, COD_Mn_ was the most critical factor, followed by NH_3_-N and TP.
4. Zooplankton community assembly in the Xichong River was dominated by deterministic processes, whereas the Jialing River exhibited seasonal variation: deterministic processes dominated in spring, shifting to stochastic processes in autumn. The integration of multiple models quantified the causal pathways linking ecosystem health status to community assembly mechanisms. An intrinsic relationship may exist between the two: pollution stress favors deterministic filtering in community assembly, whereas improved ecological conditions enhance stochastic processes, promoting community diversification.

## Figures and Tables

**Table 1:** Basic physical and chemical properties of the watershed.

Groups	NH_3_-N (mg/L)	TP (mg/L)	COD_Mn_ (mg/L)	WQI
SX	0.63 ± 0.215 ^b^	0.036 ± 0.027 ^b^	6.348 ± 0.741 ^a^	49.22 (Poor)
SJ	0.335 ± 0.282 ^c^	0.043 ± 0.034 ^b^	2.962 ± 1.536 ^b^	79.26 (Good)
AX	0.873 ± 0.347 ^a^	0.128 ± 0.038 ^a^	6.841 ± 1.119 ^a^	34.34 (Poor)
AJ	0.407 ± 0.189 ^bc^	0.055 ± 0.019 ^b^	2.592 ± 1.051 ^b^	79.78 (Good)

N represents the sample size. Data are presented as mean ± standard deviation. Means within a column are ranked from largest to smallest. Different lowercase letters indicate statistically significant differences at *p* < 0.05, while the same letter indicates no significant difference.

## Data Availability

The original contributions presented in this study are included in the article/Supplementary Materials. Further inquiries can be directed to the corresponding author.

## Supplementary Materials

The following supporting information can be downloaded at: https://www.mdpi.com/article/10.3390/biology15141211/s1.
