# Supplementary material for: Multi-Model Approaches Reveal Cascading Regulation and Ecological Responses of Zooplankton to Seasonal and Water Quality Variations in Urbanized Rivers
Source: Biology (Basel). 2026 Jul 22;15(14):1211. doi: 10.3390/biology15141211 (PMC13405743; doi:10.3390/biology15141211)
Supplement: Supplementary file 1 [file biology-15-01211-s001.zip › Supplmentary Materials.pdf]

Supplementary materials

Figure number: 4

Table number: 9

Table S1 Comprehensive evaluation results of the indices in the Jialing River Basin[12, 39, 43]

Indicator	Very Poor	Poor	Medium	Good	Excellent
Shannon-Wiener index	0	0～1	1～2	2～3	3～+∞
Pielou index	0	0～0.3	0.3～0.5	0.5～0.8	0.8～1
Margalef index	0	0～0.6	0.6～1	1～1.6	1.6～3
WQI	0～25	25～50	50～70	70～90	90～100
Z-IBI	0～19.9	19.9～39.7	39.7～59.6	59.6～79.5	79.5～100

Note: The classification criteria for diversity indices refer to Ren et al. (2011); the WQI classification refers to Pesce and Wunderlin (2000); and the relevant study by Huang et al. (2025) in the same study area further provided methodological references for the evaluation criteria used in this study.

43. Ren, L.; Zhang, Z.; Zeng, X.; Ma, Y.; Zeng, Y.; Zhou, C. Community structure of zooplankton and water quality assessment of Jialing River in Nan Chong. Procedia Environ. Sci. 2011, 10, 1321–1326.https://doi.org/10.1016/j.proenv.2011.09.211

Table S2. Spatiotemporal variations in density of zooplankton community

Groups	N	Density (Mean ± SD)ind./L
AX	12	3.5417±2.19357[a]
AJ	10	2.02±1.00089[a]
SX	12	136.233±40.52[b]
SJ	10	42.97±46.022[c]

Note: N represents the sample size. Data are presented as mean ± standard deviation. Different lowercase letters indicate statistically significant differences at $P < 0.05$, while the same letter indicates no significant difference. Specifically, the density in group SX was significantly different from those in groups AX, AJ, and SJ at $P < 0.001$.

Table S3. Candidate metrics for calculating the Z-IBI in the Jialing River Basin

Category	Candidate Metrics	Abbreviation
Species richness	Number of Protozoa species	M1
	Number of Rotifer species	M2
	Number of Cladocera species	M3
	Number of Copepoda species	M4
	Number of Total species	M5
Species density	Protozoa density	M6
	Rotifer density	M7
	Cladocera density	M8
	Copepoda density	M9
	Total density	M10
	Percentage of Protozoa density	M11
	Percentage of Rotifer density	M12
	Percentage of Cladocera density	M13
	Percentage of Copepoda density	M14
Biomass	Protozoa biomass	M15
	Rotifer biomass	M16
	Cladocera biomass	M17
	Copepoda biomass	M18
	Total biomass	M19
Diversity index	Margalef	M20
	Pielou	M21
	Shannon wiener	M22

Table S4.(1) Ecological assessment scores of sampling sites in the autumn

Site	Z-IBI	IBI health rating	WQI
AX1	37.22	Poor	48.51
AX2	40.00	Medium	35.61
AX3	49.83	Medium	41.63
AX4	40.00	Medium	47.59
AX5	18.02	Very poor	57.26
AX6	0.00	Very poor	18.39
AX7	46.33	Medium	34.34
AX8	0.38	Very poor	37.78
AX9	31.39	Poor	11.23
AX10	20.44	Poor	18.68
AX11	2.35	Very poor	34.52
AX12	36.71	Poor	26.53
AJ13	79.37	Good	83.66
AJ14	79.49	Excellent	88.10
AJ15	78.47	Good	79.26
AJ16	77.57	Good	94.46
AJ17	59.85	Good	87.70
AJ18	74.88	Good	83.28
AJ19	80.35	Excellent	81.91
AJ20	27.86	Poor	62.67
AJ21	49.82	Medium	74.86
AJ22	48.31	Medium	61.90

Table S4.(2) Ecological assessment scores of sampling sites in the spring

Site	Z-IBI	IBI health rating	WQI
SX1	11.01	Very poor	50.32
SX2	14.53	Very poor	46.77
SX3	23.02	Poor	57.01
SX4	16.99	Very poor	58.43
SX5	0.00	Very poor	53.69
SX6	3.59	Very poor	42.27
SX7	0.10	Very poor	47.16
SX8	3.03	Very poor	43.41
SX9	22.54	Poor	36.19
SX10	40.38	Medium	50.53
SX11	37.25	Poor	53.21
SX12	41.55	Medium	51.64
SJ13	45.57	Medium	83.98
SJ14	36.62	Poor	88.39
SJ15	30.03	Poor	89.97
SJ16	43.48	Medium	93.44
SJ17	61.16	Good	80.95
SJ18	54.01	Medium	88.99
SJ19	98.36	Excellent	80.33
SJ20	19.32	Very poor	50.75
SJ21	78.46	Good	51.71
SJ22	62.52	Good	84.06

Table S5.(1) Mantel test results of zooplankton communities in the Jialing River Basin(AX)

Group	spec	env	r	p	p_label	r-abs-cut
AX	Protozoa	NH_3-N	-0.044521095	0.564	ns	(0,0.4]
	Protozoa	TP	-0.133672672	0.692	ns	(0,0.4]
	Protozoa	COD_{Mn}	-0.122006317	0.622	ns	(0,0.4]
	Protozoa	Margalef	-0.173063527	0.845	ns	(0,0.4]
	Protozoa	Pielou	0.009366675	0.448	ns	(0,0.4]
	Protozoa	Shannon-Wiener	0.004594561	0.457	ns	(0,0.4]
	Rotifer	NH_3-N	-0.130455457	0.776	ns	(0,0.4]
	Rotifer	TP	-0.143759348	0.681	ns	(0,0.4]
	Rotifer	COD_{Mn}	0.44724393	0.163	ns	(0.4,0.6]
	Rotifer	Margalef	-0.163667321	0.819	ns	(0,0.4]
	Rotifer	Pielou	0.701366588	0.001		(0.6,0.8]
	Rotifer	Shannon-Wiener	0.53220237	0.002		(0.4,0.6]
	Cladocera	NH_3-N	-0.111967303	0.78	ns	(0,0.4]
	Cladocera	TP	-0.111675872	0.676	ns	(0,0.4]
	Cladocera	COD_{Mn}	-0.131330873	0.701	ns	(0,0.4]
	Cladocera	Margalef	0.147409845	0.164	ns	(0,0.4]
	Cladocera	Pielou	-0.126131495	0.703	ns	(0,0.4]
	Cladocera	Shannon-Wiener	0.181396399	0.111	ns	(0,0.4]
	Copepoda	NH_3-N	-0.118573805	0.676	ns	(0,0.4]
	Copepoda	TP	-0.041676408	0.395	ns	(0,0.4]
	Copepoda	COD_{Mn}	0.498124667	0.119	ns	(0.4,0.6]
	Copepoda	Margalef	0.042033715	0.35	ns	(0,0.4]
	Copepoda	Pielou	0.549137671	0.029		(0.4,0.6]
	Copepoda	Shannon-Wiener	0.405465947	0.059	ns	(0.4,0.6]

Note: The Pearson correlation coefficient (*r*) is used to measure the strength and direction of the linear relationship between two environmental factors or diversity indices, ranging from –1 to 1: $r > 0$ indicates a positive correlation (both variables increase or decrease together), $r < 0$ indicates a negative correlation (one

increases while the other decreases), and the larger the |*r*|, the stronger the linear association. The corresponding *P* value determines whether the correlation is statistically significant ($P < 0.05$ indicates significance; otherwise, the correlation may be due to random fluctuation). The Mantel test, based on distance matrices, evaluates the overall correlation between environmental factors and zooplankton community structure. Its core metric, Mantel's *r*, also ranges from –1 to 1: $r > 0$ suggests that greater environmental differences are associated with greater biological community differences (indicating environmental filtering dominance); $r \approx 0$ indicates no association; $r < 0$ implies that greater environmental differences are associated with smaller biological community differences (a scenario often observed when organisms respond more strongly to unmeasured factors or when the community has undergone widespread degradation). The larger the absolute value of Mantel's r, the stronger the shaping effect of the environment on community structure. Similar to Pearson, the *P* value of the Mantel test is used to judge the significance of the correlation.Pearson.

Table S5.(2) Mantel test results of zooplankton communities in the Jialing River Basin(SX)

Group	spec	env	r	p	p_label	r-abs-cut
	Protozoa	NH_3-N	-0.04223688	0.473	ns	(0,0.4]
	Protozoa	TP	-0.07714646	0.578	ns	(0,0.4]
	Protozoa	COD_{Mn}	-0.05038381	0.626	ns	(0,0.4]
	Protozoa	Margalef	-0.10943109	0.687	ns	(0,0.4]
	Protozoa	Pielou	0.18426331	0.107	ns	(0,0.4]
	Protozoa	Shannon-Wiener	0.07206772	0.326	ns	(0,0.4]
	Rotifer	NH_3-N	-0.19906779	0.946	ns	(0,0.4]
	Rotifer	TP	-0.1710135	0.867	ns	(0,0.4]
	Rotifer	COD_{Mn}	0.0757293	0.267	ns	(0,0.4]
	Rotifer	Margalef	-0.15313425	0.766	ns	(0,0.4]
	Rotifer	Pielou	0.70159537	0.001		(0.6,0.8]
SX	Rotifer	Shannon-Wiener	0.65321143	0.001		(0.6,0.8]
	Cladocera	NH_3-N	-0.16710118	0.885	ns	(0,0.4]
	Cladocera	TP	-0.1462477	0.735	ns	(0,0.4]
	Cladocera	COD_{Mn}	0.29233699	0.018		(0,0.4]
	Cladocera	Margalef	-0.19491575	0.804	ns	(0,0.4]
	Cladocera	Pielou	0.04736657	0.269	ns	(0,0.4]
	Cladocera	Shannon-Wiener	0.01898207	0.324	ns	(0,0.4]
	Copepoda	NH_3-N	-0.14937037	0.787	ns	(0,0.4]
	Copepoda	TP	-0.16342725	0.772	ns	(0,0.4]
	Copepoda	COD_{Mn}	0.18985289	0.062	ns	(0,0.4]
	Copepoda	Margalef	-0.03923025	0.44	ns	(0,0.4]
	Copepoda	Pielou	0.24715271	0.085	ns	(0,0.4]
	Copepoda	Shannon-Wiener	0.24927144	0.187	ns	(0,0.4]

Table S5.(3) Mantel test results of zooplankton communities in the Jialing River Basin(AJ)

Group	spec	env	r	p	p_label	r-abs-cut
	Protozoa	NH_3-N	0.15837087	0.261	ns	(0,0.4]
	Protozoa	TP	-0.21680555	0.755	ns	(0,0.4]
	Protozoa	COD_{Mn}	0.35685395	0.052	ns	(0,0.4]
	Protozoa	Margalef	0.02857569	0.369	ns	(0,0.4]
	Protozoa	Pielou	-0.07214792	0.591	ns	(0,0.4]
	Protozoa	Shannon-Wiener	0.02255191	0.418	ns	(0,0.4]
	Rotifer	NH_3-N	0.10663412	0.169	ns	(0,0.4]
	Rotifer	TP	0.42406057	0.113	ns	(0.4,0.6]
	Rotifer	COD_{Mn}	0.35240869	0.08	ns	(0,0.4]
	Rotifer	Margalef	0.11595038	0.216	ns	(0,0.4]
	Rotifer	Pielou	0.18538859	0.169	ns	(0,0.4]
AJ	Rotifer	Shannon-Wiener	0.08588303	0.296	ns	(0,0.4]
	Cladocera	NH_3-N	0.10513855	0.177	ns	(0,0.4]
	Cladocera	TP	0.04197217	0.302	ns	(0,0.4]
	Cladocera	COD_{Mn}	-0.18205043	0.865	ns	(0,0.4]
	Cladocera	Margalef	-0.04790615	0.552	ns	(0,0.4]
	Cladocera	Pielou	-0.08050734	0.606	ns	(0,0.4]
	Cladocera	Shannon-Wiener	-0.13404034	0.768	ns	(0,0.4]
	Copepoda	NH_3-N	-0.01661758	0.379	ns	(0,0.4]
	Copepoda	TP	0.33563869	0.087	ns	(0,0.4]
	Copepoda	COD_{Mn}	0.14499744	0.204	ns	(0,0.4]
	Copepoda	Margalef	0.27245004	0.122	ns	(0,0.4]
	Copepoda	Pielou	0.32445959	0.054	ns	(0,0.4]
	Copepoda	Shannon-Wiener	0.14819291	0.202	ns	(0,0.4]

Table S5.(4) Mantel test results of zooplankton communities in the Jialing River Basin(SJ)

Group	spec	env	r	p	p_label	r-abs-cut
	Protozoa	NH_3-N	-0.16945317	0.811	ns	(0,0.4]
	Protozoa	TP	-0.17026417	0.694	ns	(0,0.4]
	Protozoa	COD_{Mn}	-0.1361987	0.643	ns	(0,0.4]
	Protozoa	Margalef	0.11590564	0.27	ns	(0,0.4]
	Protozoa	Pielou	-0.11861127	0.731	ns	(0,0.4]
	Protozoa	Shannon-Wiener	-0.13223425	0.684	ns	(0,0.4]
	Rotifer	NH_3-N	0.43633162	0.155	ns	(0.4,0.6]
	Rotifer	TP	0.05055209	0.318	ns	(0,0.4]
	Rotifer	COD_{Mn}	-0.07177249	0.401	ns	(0,0.4]
	Rotifer	Margalef	0.54027001	0.01		(0.4,0.6]
	Rotifer	Pielou	0.38550841	0.054	ns	(0,0.4]
SJ	Rotifer	Shannon-Wiener	-0.19764423	0.959	ns	(0,0.4]
	Cladocera	NH_3-N	0.02340509	0.315	ns	(0,0.4]
	Cladocera	TP	-0.10340404	0.594	ns	(0,0.4]
	Cladocera	COD_{Mn}	-0.19805376	0.919	ns	(0,0.4]
	Cladocera	Margalef	0.26918603	0.105	ns	(0,0.4]
	Cladocera	Pielou	0.02128217	0.344	ns	(0,0.4]
	Cladocera	Shannon-Wiener	-0.20080857	0.935	ns	(0,0.4]
	Copepoda	NH_3-N	-0.18291889	0.925	ns	(0,0.4]
	Copepoda	TP	-0.03883698	0.411	ns	(0,0.4]
	Copepoda	COD_{Mn}	-0.16689642	0.85	ns	(0,0.4]
	Copepoda	Margalef	-0.05136535	0.498	ns	(0,0.4]
	Copepoda	Pielou	0.14027736	0.231	ns	(0,0.4]
	Copepoda	Shannon-Wiener	-0.11140956	0.716	ns	(0,0.4]

Table S6. Environmental Quality Standards for Surface Water of the People's Republic of China (GB 3838—2002)

Index (mg/L)	Class I	Class II	Class III	Class IV	Class V
COD_{Mn}	≤ 2	≤ 4	≤ 6	≤ 10	≤ 15
NH_3-N	≤ 0.15	≤ 0.5	≤ 1.0	≤ 1.5	≤ 2.0
TP	≤ 0 .02	≤ 0.1	≤ 0.2	≤ 0.3	≤ 0.4

Table S7.(1) Water quality parameter values of sampling sites across the study area(Spring)

Sample	NH_3-N (mg/L)	TP (mg/L)	COD_{Mn} (mg/L)
SX1	0.567	0.029	6.436
SX2	0.633	0.016	6.874
SX3	0.387	0.031	5.973
SX4	0.425	0.024	5.750
SX5	0.43	0.01	6.519
SX6	0.666	0.026	7.303
SX7	0.441	0.007	7.407
SX8	0.644	0.016	7.303
SX9	1.175	0.096	6.125
SX10	0.732	0.056	5.715
SX11	0.721	0.058	5.360
SX12	0.748	0.067	5.415
SJ1	0.261	0.117	1.792
SJ2	0.255	0.037	1.992
SJ3	0.321	0.014	1.833
SJ4	0.135	0.014	1.842
SJ5	0.146	0.018	3.472
SJ6	0.135	0.018	2.408
SJ7	0.233	0.039	3.125
SJ8	1.093	0.09	4.418

SJ9	0.387	0.043	6.575
SJ10	0.392	0.043	2.167

Table S7.(2) Water quality parameter values of sampling sites across the study area(Autumn)

Sample	NH_3-N (mg/L)	TP (mg/L)	COD_{Mn} (mg/L)
AX1	0.521	0.105	6.056
AX2	0.605	0.109	7.551
AX3	0.593	0.105	6.804
AX4	0.622	0.091	6.056
AX5	0.605	0.114	4.561
AX6	0.844	0.132	9.047
AX7	0.706	0.100	7.551
AX8	0.742	0.119	6.804
AX9	1.334	0.229	7.801
AX10	1.442	0.165	7.137
AX11	1.035	0.128	6.400
AX12	1.430	0.142	6.326
AJ1	0.353	0.096	1.804
AJ2	0.294	0.050	1.804
AJ3	0.455	0.050	2.588
AJ4	0.282	0.045	1.020
AJ5	0.353	0.040	1.804
AJ6	0.282	0.040	2.588
AJ7	0.335	0.045	2.588
AJ8	0.497	0.082	4.419
AJ9	0.311	0.040	3.657
AJ10	0.904	0.063	3.657

Table S8.(1) One-way ANOVA for diversity indices (Margalef)

(I) Group	(J) Group	Mean Difference (I-J)	Std. Error	P	95% Confidence Interval	
					Lower Bound	Upper Bound
AX	AJ	-0.45467	0.31668	0.159	-1.0947	0.1854
	SX	-0.51583	0.30195	0.095	-1.1261	0.0944
	SJ	0.01133	0.31668	0.972	-0.6287	0.6514
AJ	AX	0.45467	0.31668	0.159	-0.1854	1.0947
	SX	-0.06117	0.31668	0.848	-0.7012	0.5789
	SJ	0.466	0.33076	0.167	-0.2025	1.1345
SX	AX	0.51583	0.30195	0.095	-0.0944	1.1261
	AJ	0.06117	0.31668	0.848	-0.5789	0.7012
	SJ	0.52717	0.31668	0.104	-0.1129	1.1672
SJ	AX	-0.01133	0.31668	0.972	-0.6514	0.6287
	AJ	-0.466	0.33076	0.167	-1.1345	0.2025
	SX	-0.52717	0.31668	0.104	-1.1672	0.1129

Group	N	Mean	Std. Deviation	Std. Error	95% Confidence Interval		Min	Max
					Lower Bound	Upper Bound		
AX	12	3.4033	1.00113	0.289	2.7672	4.0394	1.52	4.84
AJ	10	3.858	0.48896	0.15462	3.5082	4.2078	3.24	4.82
SX	12	3.9192	0.58931	0.17012	3.5447	4.2936	2.84	4.89
SJ	10	3.392	0.73669	0.23296	2.865	3.919	2.37	4.73
Total	44	3.6448	0.75603	0.11398	3.4149	3.8746	1.52	4.89

Note: All statistical analyses were performed using SPSS 27. One-way ANOVA was used to test overall differences among the four groups (SJ, SX, AJ, AX) for each diversity index, followed by Tukey HSD post-hoc tests for pairwise comparisons. Prior to ANOVA, homogeneity of variances was verified using Levene's test (all $P > 0.05$). The significance level was set at $\alpha = 0.05$. The table reports group means, standard deviations (SD), standard errors (SE), 95% confidence intervals (CI), ANOVA F-statistics with degrees of freedom, and Tukey HSD pairwise comparison results. Groups sharing the same letter are not significantly different ($P \geq 0.05$). Abbreviations: AX, Xichong River autumn; AJ, Jialing River autumn; SX, Xichong River spring; SJ, Jialing River spring; ns, not significant.

Table S8.(2) One-way ANOVA for diversity indices (Shannon Wiener)

(I) Group	(J) Group	Mean Difference (I-J)	Std. Error	P	95% Confidence Interval	
					Lower Bound	Upper Bound
AX	AJ	-0.2985	0.16522	0.078	-0.6324	0.0354
	SX	-0.36250*	0.15753	0.027	-0.6809	-0.0441
	SJ	-0.51650*	0.16522	0.003	-0.8504	-0.1826
AJ	AX	0.2985	0.16522	0.078	-0.0354	0.6324
	SX	-0.064	0.16522	0.701	-0.3979	0.2699
	SJ	-0.218	0.17256	0.214	-0.5668	0.1308
SX	AX	0.36250*	0.15753	0.027	0.0441	0.6809
	AJ	0.064	0.16522	0.701	-0.2699	0.3979
	SJ	-0.154	0.16522	0.357	-0.4879	0.1799
SJ	AX	0.51650*	0.16522	0.003	0.1826	0.8504
	AJ	0.218	0.17256	0.214	-0.1308	0.5668
	SX	0.154	0.16522	0.357	-0.1799	0.4879

Group	N	Mean	Std. Deviation	Std. Error	95% Confidence Interval		Min	Max
					Lower Bound	Upper Bound		
AX	12	2.1625	0.38407	0.11087	1.9185	2.4065	1.47	2.59
AJ	10	2.461	0.39433	0.1247	2.1789	2.7431	1.89	3.08
SX	12	2.525	0.47126	0.13604	2.2256	2.8244	1.74	3.26
SJ	10	2.679	0.23345	0.07382	2.512	2.846	2.28	2.99
Total	44	2.4466	0.41875	0.06313	2.3193	2.5739	1.47	3.26

Table S8.(3) One-way ANOVA for diversity indices (Pielou)

(I) Group	(J) Group	Mean Difference (I-J)	Std. Error	P	95% Confidence Interval	
					Lower Bound	Upper Bound
AX	AJ	-0.03933	0.05111	0.446	-0.1426	0.064
	SX	-0.03167	0.04874	0.52	-0.1302	0.0668
	SJ	-0.12133*	0.05111	0.022	-0.2246	-0.018
AJ	AX	0.03933	0.05111	0.446	-0.064	0.1426
	SX	0.00767	0.05111	0.882	-0.0956	0.111
	SJ	-0.082	0.05339	0.132	-0.1899	0.0259
SX	AX	0.03167	0.04874	0.52	-0.0668	0.1302
	AJ	-0.00767	0.05111	0.882	-0.111	0.0956
	SJ	-0.08967	0.05111	0.087	-0.193	0.0136
SJ	AX	0.12133*	0.05111	0.022	0.018	0.2246
	AJ	0.082	0.05339	0.132	-0.0259	0.1899
	SX	0.08967	0.05111	0.087	-0.0136	0.193

Group	N	Mean	Std. Deviation	Std. Error	95% Confidence Interval		Min	Max
					Lower Bound	Upper Bound		
AX	12	0.7467	0.13186	0.03807	0.6629	0.8304	0.48	0.95
AJ	10	0.786	0.11909	0.03766	0.7008	0.8712	0.57	0.94
SX	12	0.7783	0.1355	0.03912	0.6922	0.8644	0.54	0.94
SJ	10	0.868	0.07391	0.02337	0.8151	0.9209	0.74	0.95
Total	44	0.7918	0.12343	0.01861	0.7543	0.8293	0.48	0.95

Table S9. Classification criteria for disturbance types

Disturbance Type	Criteria
Natural river channel	Within a 500 m radius around the site: proportion of cropland < 10%, proportion of built-up land < 5%; continuous natural vegetation coverage along the riparian zone > 70%; no identifiable sewage outfalls; no dams, sand mining, or other engineering facilities.
Urban disturbance	Within a 500 m radius around the site: proportion of built-up land > 30%; presence of ≥ 2 visible sewage outfalls (including signs of domestic sewage and industrial wastewater discharge); proportion of hardened riparian zone > 50%.
Agricultural reclamation disturbance	Within a 500 m radius around the site: proportion of cropland > 40%; natural vegetation along the riparian zone fragmented, with continuous coverage < 30%; no concentrated urban built-up areas; no large-scale water conservancy facilities.
Dammed river disturbance	The site is located within 1 km upstream or downstream of a dam; the dam structure causes significant alteration of flow regime (reduced flow velocity upstream or flow reduction/dewatering downstream).
Engineering disturbance	Presence of large-scale under-construction or existing water-related engineering projects (e.g., bridge construction, embankment construction) in the vicinity of the site, with construction disturbance extending to the riparian zone.
Tributary disturbance	The site is located within 1 km upstream or downstream of the confluence of the Jialing River mainstream and a major tributary, and is directly influenced by the inflow of water, sediment, and water quality from the tributary.
Sand mining disturbance	Within a 500 m radius around the site: presence of active sand mining operations or historical sand mining sites; visible disturbance of riverbed substrate.

Notes: Prior to formal sampling, the research team conducted three rounds of field reconnaissance of all candidate sites along the Jialing River mainstream in the Nanchong section (301 km) and the entire Xichong River basin (121 km). Each reconnaissance was carried out by at least two researchers, who recorded site coordinates, conducted walking surveys along the riparian zone, and took field photographs. The surveys covered land use types within a 500 m radius around each site (proportions of cropland, built-up land, forest land, etc.), proportion of hardened riparian zone, number and scale of sewage outfalls, and the distribution of engineering facilities such as dams and sand mining operations. Three experts (with backgrounds in aquatic ecology and over 10 years of experience in river ecological assessment) independently classified all 22 sites based on the above criteria, combined with reconnaissance records and field photographs. The results showed that the classifications were

essentially identical among the three experts for all 22 sites. The final site classifications in this study were determined through joint discussion by the three experts. The classification criteria are operable and reproducible.

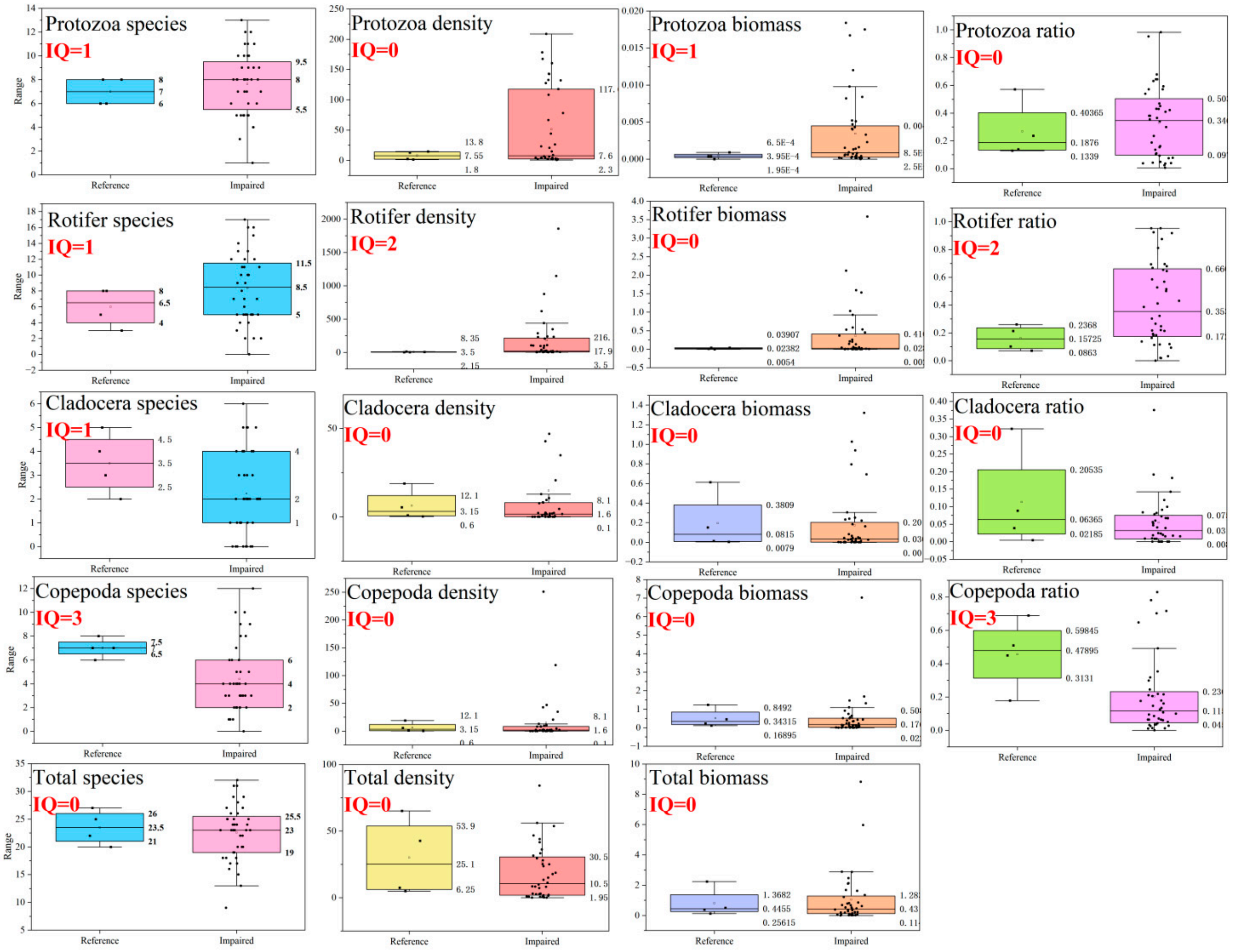

Figure S1. Boxplots of the interquartile range (IQR) for candidate metrics in the Z-IBI assessment. For each candidate metric, boxplots of reference sites (n = 4) and disturbed sites (n = 18) were plotted separately to compare the overlap of the 25%–75% interquartile ranges (boxes) between the two groups, with scores assigned according to the standardized IQ scoring framework: IQ = 0 — medians of both groups fall within each other's boxes (almost no discriminant ability); IQ = 1 — boxes partially overlap, with only one group's median falling outside the other's box (weak discriminant ability); IQ = 2 — boxes partially overlap, with medians of both groups falling outside each other's boxes (good discriminant ability, indicating substantial separation in central tendency between reference and disturbed sites); IQ = 3 — boxes do not overlap at all, with medians of both groups falling outside each other's boxes (excellent discriminant ability). Metrics with IQ ≥ 2 were retained for subsequent analysis, as this threshold ensures reliable capacity to distinguish between reference and disturbed sites.

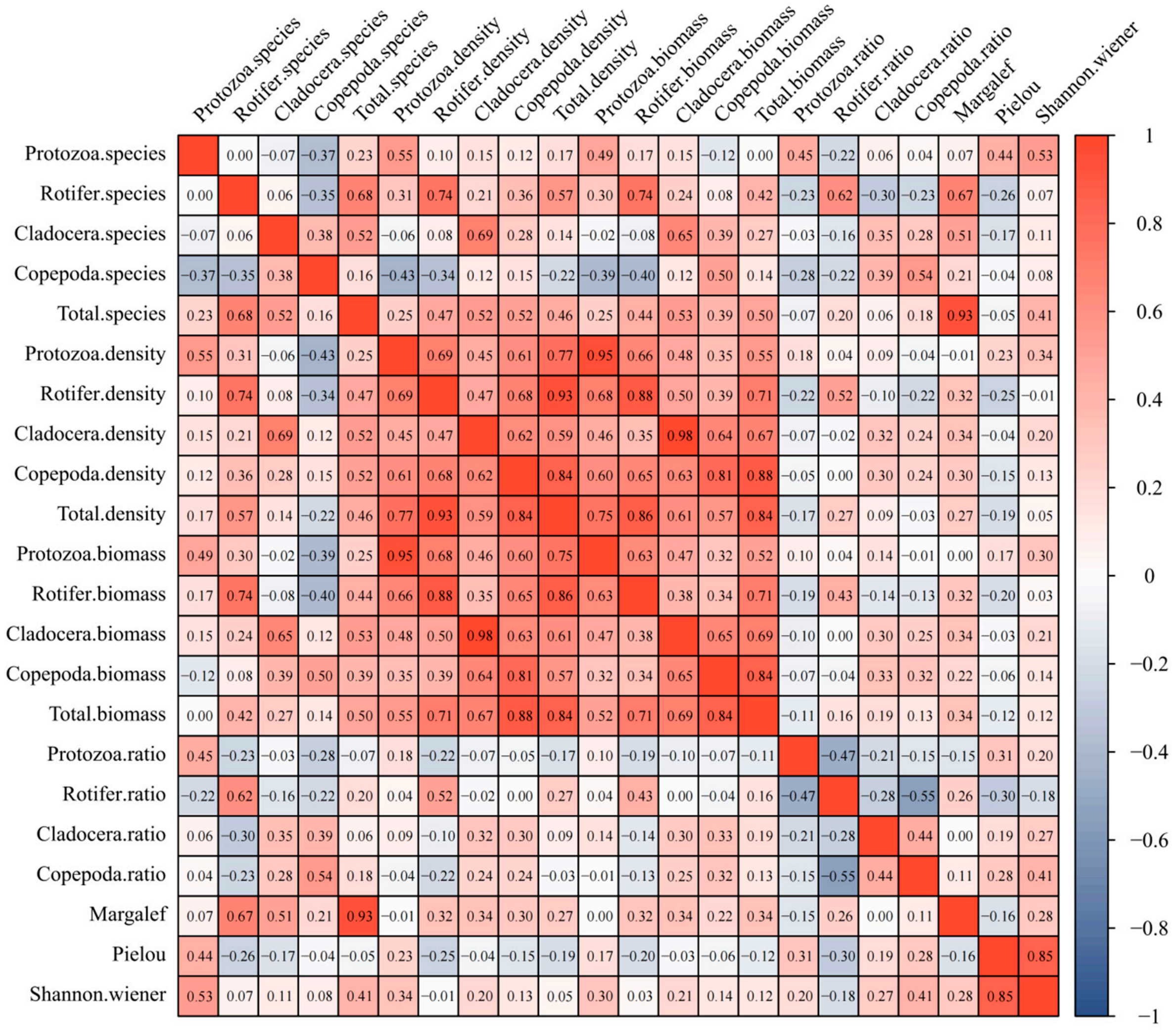

Figure S2. Spearman correlation analysis of candidate metrics for Z-IBI assessment in the Jialing River Basin

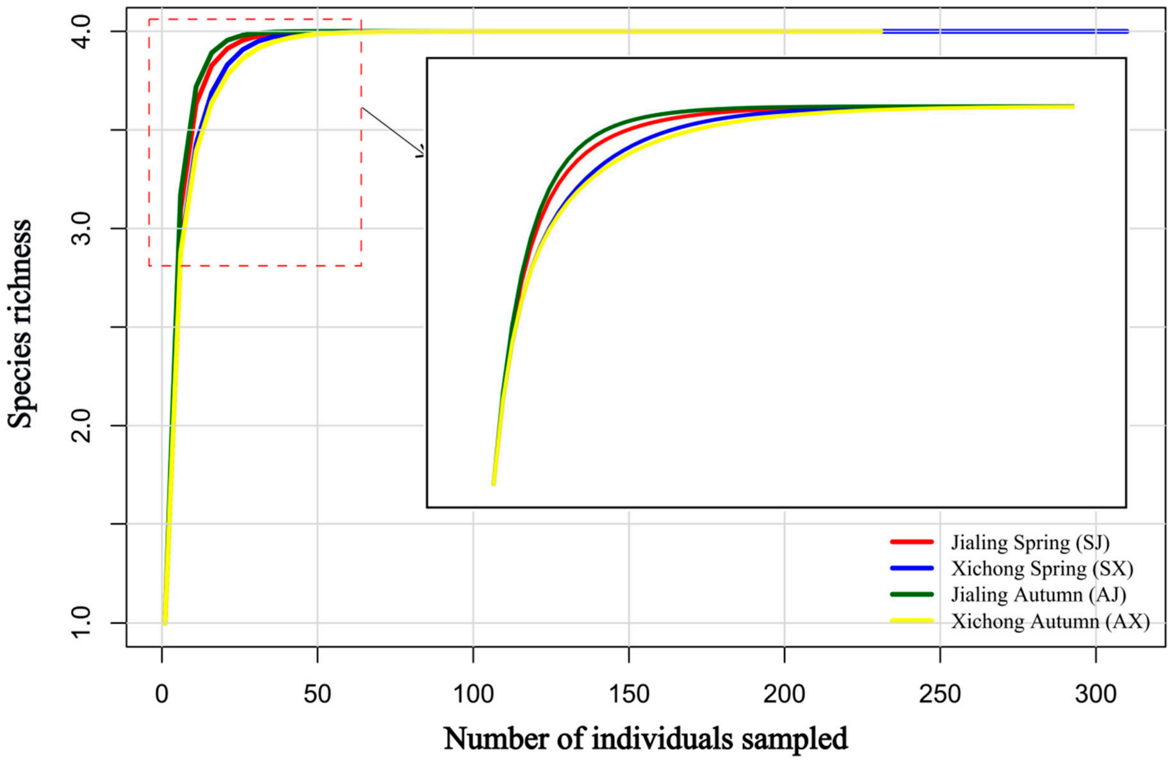

Figure S3. Rarefaction curves of zooplankton communities across four groups (SJ, SX, AJ, AX) in the Jialing River Basin. The x-axis represents the number of individuals sampled, and the y-axis represents species richness (number of taxa detected, maximum = 4). Red: Jialing Spring (SJ); Blue: Xichong Spring (SX); Green: Jialing Autumn (AJ); Yellow: Xichong Autumn (AX). All curves leveled off after approximately 50–80 individuals, indicating that sampling effort was sufficient to capture the dominant taxa in each group. The inset provides a zoomed-in view of the curve trajectories during the initial sampling stage.

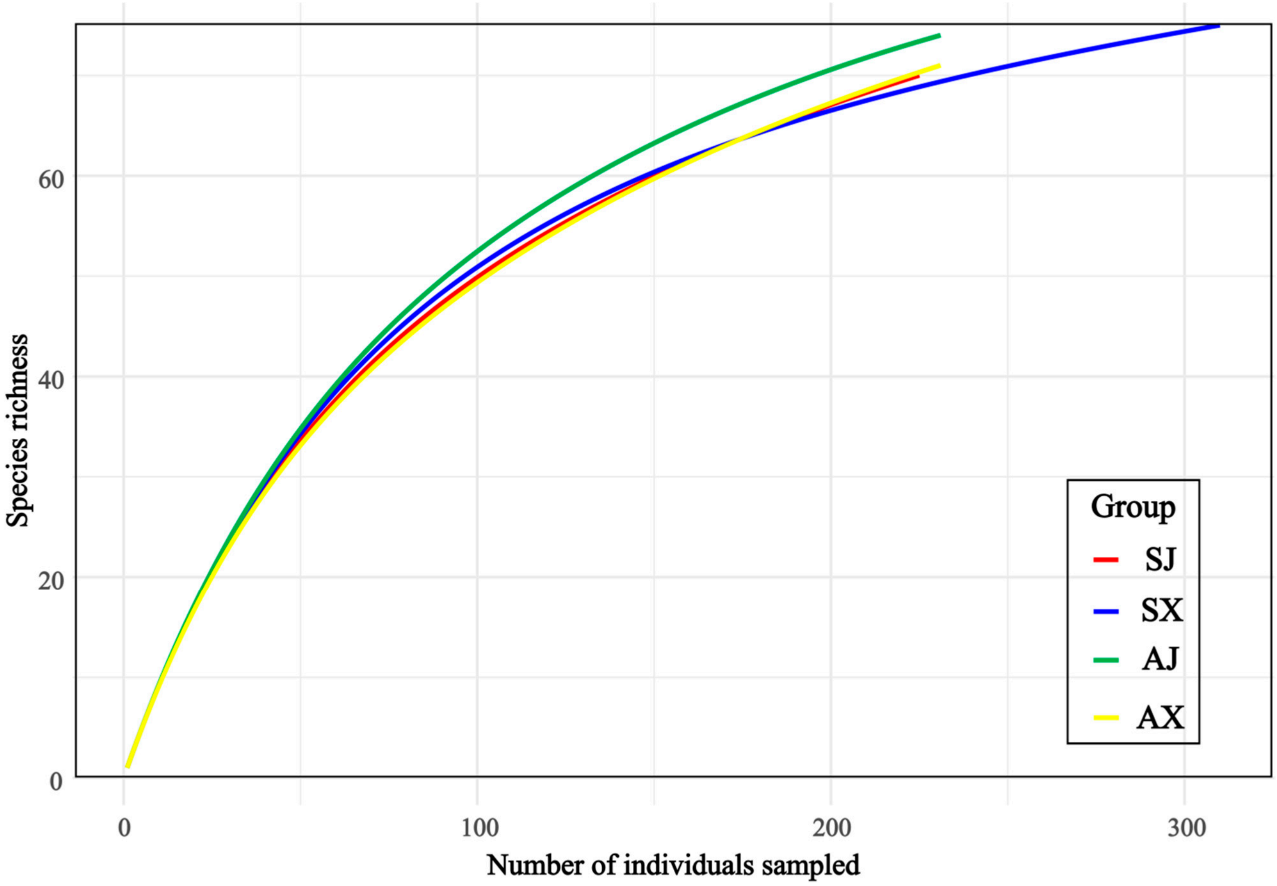

Figure S4. Rarefaction curves based on zooplankton species-level data. Based on species-level identification data, rarefaction curves for each group were generated following the same method as in Figure S3. Color coding is consistent with Figure S3. Although the absolute number of species was relatively limited due to stringent morphological identification criteria, all four curves exhibited clear inflections, confirming that sampling depth at the species level was also sufficient. However, since the overall species collected differed across seasons and river systems, the final species richness was not used as the sole analytical result.

To further verify the adequacy and reliability of sampling, this study supplemented the analysis with individual-based rarefaction analysis. Rarefaction curves were generated based on the four major groups (Protozoa, Rotifera, Cladocera, and Copepoda) and species-level taxonomic units, respectively (Figures S3 and S4). The results showed that at both taxonomic levels, the cumulative number of species/groups in all curves increased rapidly with increasing numbers of individuals sampled and leveled off after reaching a certain sampling effort. This indicates that the sampling depth in this study was sufficient to capture the major zooplankton groups and common species in each habitat, and that the subsequent multivariate statistical analyses are robust. Further comparison of rarefaction curves among groups revealed that, at the four-group level, the plateau values of group accumulation in the Jialing River mainstream (AJ, SJ) were higher than those in the Xichong River (AX, SX), and required fewer individuals to reach the plateau. This suggests that the distribution of the four major zooplankton

groups was more even in the mainstream, with higher community complexity and stability. At the species level, the analysis further showed that the Xichong River in autumn (AX) exhibited the largest initial slope, indicating the possible presence of strong dominant species in this community, which is consistent with the aforementioned finding that rotifers became the absolutely dominant group in the tributary.
